# Single cell census of human kidney organoids shows reproducibility and diminished off-target cells after transplantation

**DOI:** 10.1038/s41467-019-13382-0

**Published:** 2019-11-29

**Authors:** Ayshwarya Subramanian, Eriene-Heidi Sidhom, Maheswarareddy Emani, Katherine Vernon, Nareh Sahakian, Yiming Zhou, Maria Kost-Alimova, Michal Slyper, Julia Waldman, Danielle Dionne, Lan T. Nguyen, Astrid Weins, Jamie L. Marshall, Orit Rosenblatt-Rosen, Aviv Regev, Anna Greka

**Affiliations:** 1 Broad Institute of MIT and Harvard, Cambridge, MA USA; 2 Department of Medicine, Brigham and Women’s Hospital and Harvard Medical School, Boston, MA USA; 3 Center for the Development of Therapeutics, Broad Institute of MIT and Harvard, Cambridge, MA USA; 4 Department of Pathology, Brigham and Women’s Hospital and Harvard Medical School, Boston, MA USA; 5 Department of Biology, Howard Hughes Medical Institute, Massachusetts Institute of Technology, Cambridge, MA USA

**Keywords:** Cellular signalling networks, Stem-cell differentiation, Kidney

## Abstract

Human iPSC-derived kidney organoids have the potential to revolutionize discovery, but assessing their consistency and reproducibility across iPSC lines, and reducing the generation of off-target cells remain an open challenge. Here, we profile four human iPSC lines for a total of 450,118 single cells to show how organoid composition and development are comparable to human fetal and adult kidneys. Although cell classes are largely reproducible across time points, protocols, and replicates, we detect variability in cell proportions between different iPSC lines, largely due to off-target cells. To address this, we analyze organoids transplanted under the mouse kidney capsule and find diminished off-target cells. Our work shows how single cell RNA-seq (scRNA-seq) can score organoids for reproducibility, faithfulness and quality, that kidney organoids derived from different iPSC lines are comparable surrogates for human kidney, and that transplantation enhances their formation by diminishing off-target cells.

## Abstract (web summary)

How reproducible human kidney organoids derived from different iPSC lines are, and how faithful they are to human kidney tissue remain unclear. Here, the authors use four human iPSC lines to derive kidney organoids and show how organoid composition is reproducible, comparable to human tissue and of improved quality after transplantation.

## Introduction

Kidney diseases affect ~800 million people worldwide^1^. Despite the enormous disease burden, therapeutic innovation has lagged^2^, owing in part to the lack of appropriate models that reflect the cellular complexity of the human kidney. Technologies to generate kidney organoids from patient-derived induced pluripotent stem cells (iPSC)^3–13^—more so than generic organoids from embryonic stem cells (ESC)—promise to advance our understanding of disease-specific pathways and expedite mechanism-based therapeutic development.

To harness the full potential of iPSC-derived kidney organoid technology, we must address critical unsolved questions about their reproducibility, faithfulness and quality. First, we must establish organoid comparability at the single-cell level across several human iPSC lines. While previous efforts include scRNA-seq profiling of organoids from one^14^ or two ESC lines^15^, from one iPSC line^16^, between one iPSC and one ESC line^17^, as well as bulk RNA-seq comparing organoids from two iPSC lines^18^, no study to date has directly compared organoids from multiple iPSC lines at the single-cell level. We must also define organoid faithfulness: how well organoids from different iPSC lines recapitulate kidney development and disease-associated genes at single-cell resolution. Finally, we must address organoid quality and in particular, we must identify ways to remove off-target cells^16–18^. A comprehensive single-cell survey of kidney organoids from several iPSC lines is thus critically needed, as individual patient iPSCs become essential tools for precision medicine and drug development projects^7,19,20^.

Here, we address these critical questions by surveying organoid composition from more than 450,000 single cells across several iPSC lines, multiple replicates, differentiation protocols, developmental time, and after organoid transplantation into mouse, benchmarked against human fetal and adult kidney.

## Results

### 49 kidney organoid states from four human iPSC lines for a total of 450 K cells

**Fig. 1:**
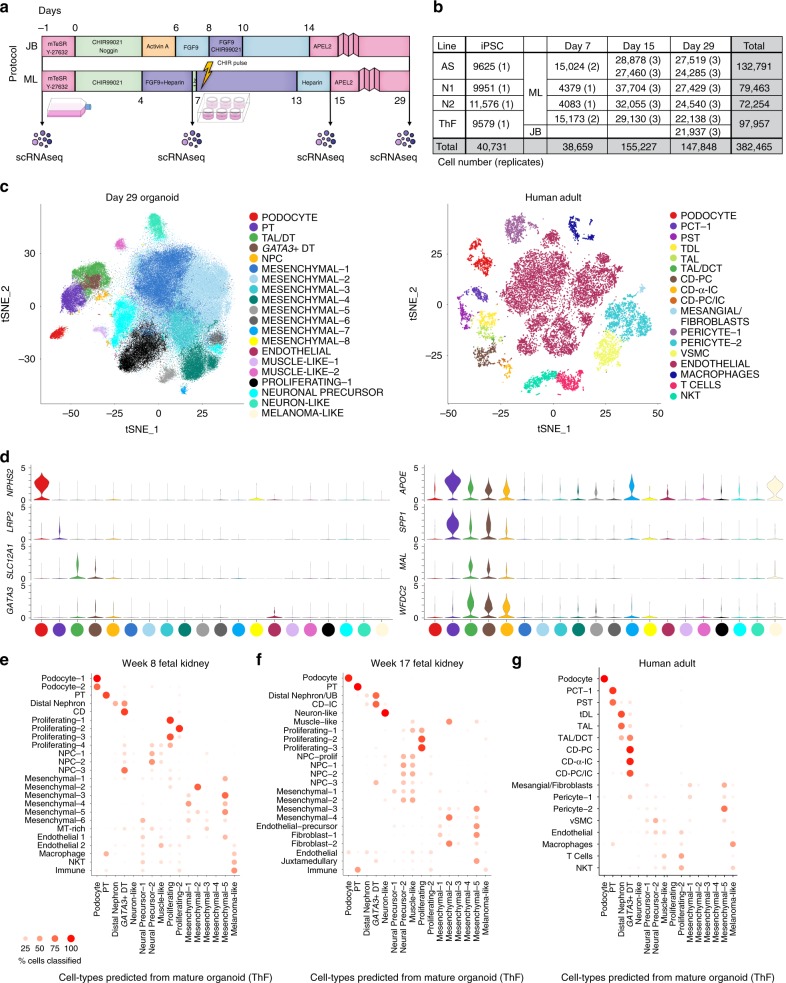
Mature kidney organoids from four different human iPSC lines contain major nephron cell classes. **a** Schematic of differentiation protocols for kidney organoids derived from human iPSCs by the JB and ML protocols (methods). Single-cell sequencing time-points as shown. **b** Table summarizing single cells profiled across four different human iPSC cell lines (AS, N1, N2, ThF) using two different protocols (ML, JB) across four time points (iPSC, Day 7 (D7), 15 (D15) and 29 (D29)). Replicates are indicated in parentheses. **c** t-SNE plot of composite single-cell transcriptomic profiles from all four iPSC D29 kidney organoids (left) and human adult kidney (right). Cluster color annotations as shown. **d** (left) Violin plots showing expression of canonical adult human kidney markers used to identify nephron clusters in D29 organoids: podocyte (*NPHS2*), proximal tubule (PT; *LRP2*), thick ascending limb/distal nephron (TAL; *SLC12A1*) and immature distal nephron (*GATA3* + DT). Mesenchymal and off-target cells were also detected. (right) Violin plots of data-derived tubular markers revealed proximal to distal gradient of *APOE* expression, proximal marker *SPP1*, and distal markers *WFDC2* and *MAL*. **e** Random forest classifier trained on D29 ML_ThF organoid gene expression profiles accurately predicted respective nephron cell classes in human first and second trimester fetal kidney (**e**, **f**) as compared to adult kidney (**g**). The *Y*-axis labels in each panel represent the annotated cell class in each human sample. The X-axis labels are predicted cell classes at D29 (ML_ThF organoids). The size and intensity of the red dot represents the % of cells in the *Y*-axis cell-types classified as the corresponding *X*-axis cell-class, e.g., all human adult podocytes are correctly classified as podocytes, while distal convoluted tubule (DCT) is predicted to be most similar to the *GATA3* *+* DT organoid cell class.

To compare organoids at single-cell resolution, we generated them according to two protocols, from each of four different human iPSC lines (generated by different methods, episomal or Sendai virus; Supplementary Fig. 1A), and at multiple time points along differentiation. We profiled single cells by droplet-based scRNA-seq (Fig. 1a). We used the ML protocol^7^ and generated organoids from each of two commercial lines (Thermo Fisher (ThF, female) and Alstem (AS, male)) and two obtained from human healthy donors (N1, female and N2, male) (Supplementary Fig. 1A). We profiled single cells at the day 0 (D0) iPSC state, at two critical milestones, day 7 (D7; immediately prior to cells being plated for 3D self-organization and nephrogenesis), and day 15 (D15; when growth factor treatment ends), and finally, at day 29 (D29) when the organoids are mature. This experimental design allowed us to assess the reproducibility within a protocol and the impact of cell-line-specific differences in iPSC pluripotency and/or subsequent differentiation. Next, we generated kidney organoids using the JB protocol^8^ from ThF iPSCs at D29 (Fig. 1a). To account for technical variability, we sequenced single cells from three different iPSC replicates (D15, D29, all lines) and an additional, independent passage (AS line at D7, D15, D29 and TF at D7). We successfully profiled 382,465 single cells from organoids across all four iPSC lines (Fig. 1b and Supplementary Fig. 1B) and compared them to 17,759 cells profiled from the unaffected cortical and medullary kidney portions of tumor nephrectomies from three adult males (with no known kidney diseases) (Fig. 1c). We determined the cellular composition of organoids using unsupervised graph-based clustering followed by post-hoc annotation (Methods, Supplementary Data 1) with signatures of cell types (Supplementary Fig. 2, Supplementary Table 1) and cell cycle genes^21^ (Supplementary Data 2).

D29 organoids from all four iPSC lines contained cells representative of segments of a developing nephron (Fig. 1c, d and Supplementary Fig. 2): podocytes (*NPHS2*, podocin; *NPHS1*, nephrin; *SYNPO*, synaptopodin; *WT1*, Wilms Tumor 1), proximal tubular (PT) cells (*LRP2*, megalin), thick ascending limb (TAL; *SLC12A1*, Na-K-Cl cotransporter), and distal nephron cells (*CDH1*, E-cadherin; *AQP2*, aquaporin 2; *GATA3*). There was no cluster enriched for *SLC12A3* (Na-Cl symporter; Supplementary Fig. 2), a canonical marker of the distal convoluted tubule (DCT). The organoid single-cell profiles retained the proximal (podocyte) to distal axis of the human nephron (Fig. 1c, left) on visualization of the data using t-distributed stochastic nonlinear embedding (tSNE), unlike the discrete clusters seen in adult kidney (Fig. 1c, right). We identified data-derived markers (Supplementary Table 2), including osteopontin (*SPP1*)^22^ in the proximal tubular cell cluster, and *WFDC2*^23^ and *MAL*^24^ in the distal nephron cluster (Fig. 1d and Supplementary Fig. 3A), in line with scRNA-seq studies of human adult and fetal kidney^25^. In all four iPSC lines, we observed a proximal to distal tubular gradient of *APOE* (a gene associated with diabetic kidney disease^26^) (Fig. 1d). Thus, D29 organoids reproducibly developed podocytes, proximal tubular cells, and cells consistent with the TAL and distal nephron (but without a defined DCT or collecting duct (CD) segment as seen in adult kidney).

D29 organoids also contained nephron progenitor cells (NPC) enriched in *PAX2*, *LHX1* and *PAX8* (top cluster-specific differentially expressed (DE) genes). The majority of the organoid single cells (70% on average) were mesenchymal (Fig. 1c), grouped in eight subsets (Mesenchymal 1–8) enriched for markers of progenitor and differentiating cell types (Fig.1c; Supplementary Fig. 2). Prominent non-kidney off-target populations^17^, absent in adult human kidney (Fig. 1c, Supplementary Fig. 3B), were found in D29 organoids, including melanoma-like cells (*PMEL*), *SOX2*-positive(+) neuronal precursors, *STMN2*+ neuron-like cells, and *MYOG*+ muscle-like cells, as reported previously^14–18^. A rare population of endothelial cells (EC) was also observed (Fig. 1c and Supplementary Fig. 2).

We compared the cell clusters from D29 human kidney organoids (ThF line) to human tissue, specifically to fetal kidney from the first (8 weeks) and second (17 weeks) trimesters (Supplementary Fig. 4)^27,28^, and to adult kidney (Fig. 1c, right). Using a classification-based approach (random forest classifier)^29^, we assessed the correspondence between organoid and human tissue cell clusters (Fig. 1e–g, Methods).

Overall, organoid cells were most similar to first and second trimester fetal kidneys, largely consistent with previous studies^4,16,18^ (Fig. 1e, f). All nephron lineages were accurately classified by the algorithm (Fig. 1e–g). Compared to those in adult human kidney (Fig. 1c), all organoids contained cells of human nephron segments, but with poorly differentiated distal tubular cells. In contrast, in the adult kidney, we identified distinct proximal, loop of Henle (LoH), DCT and CD subclusters, including principal cells (PC) and α- and β-intercalated cells (IC), as well as endothelial and immune clusters (Fig. 1c, Supplementary Fig. 3B).

Interestingly, some of the mesenchymal cell types in kidney organoids were predictive of cell types in fetal and adult kidney, suggesting that these are on-target mesenchymal cells. Fetal kidney cell types (mesenchymal, endothelial, and fibroblasts) were classified to mesenchymal-1, -2, and -5 clusters in organoids (Fig. 1e, f, Supplementary Data 3), while mesangial cells, fibroblasts, and pericytes in adult kidney were classified to mesenchymal-1 and -5 in organoids (Fig. 1g). Of interest, mesenchymal-2 cells in organoids were enriched for *MGP*, *GAS2*, *PRXX2* and *FOXP2*^30^, known markers of fetal mesangial cells in the developing kidney. Similarly, mesenchymal-5 cells in organoids were enriched for fetal stromal genes *SULTIE1*, *DKK1*^31^, *NR2F1*^32^, *ID3*, *ZEB2*, *COL6A3*^33^ and *DCN*. In contrast, mesenchymal-4 that did not map to human kidney cell types was enriched in genes associated with cartilage formation^34^ (*ACAN*, *SOX9*, *MATN4*, *LECT1*, *EPYC*, *COL9A3* and *COL9A1*)(Fig. 1e, f).

To directly compare organoids to human kidney cells, we performed pairwise joint clustering analysis of mature organoids and human kidney (adult, Supplementary Fig. 5A, fetal trimester 1, Supplementary Fig. 5B and fetal trimester 2, Supplementary Fig. 5C). In all cases, we identified the same nephron cell types as predicted by the random forest (RF) classifier: podocytes, PT, TAL and distal nephron cells. Joint clustering of fetal and organoid cells revealed shared proliferating and nephron progenitor states absent in adult kidney (and consistent with the RF results, Fig. 1e–g). Of particular interest, the organoid GATA3+ distal tubular cells coclustered with adult CD principal cells (Supplementary Fig. 5A) and second trimester distal tubule cluster (Supplementary Fig. 5C), but not with first trimester distal tubule cluster (Supplementary Fig. 5B). While *GATA3*+ cells have been previously reported^7,16^, our analyses extend these findings by uncovering a *CDH1+*/*GATA3*+ tubular epithelial organoid cluster likely representing a developing CD principal cell structure that is more mature than first trimester kidney.

In the joint clustering analysis, the mesenchymal compartment included organoid-specific mesenchymal types (O-Mesenchymal-2, enriched for expression of *COL9* genes), adult/fetal-specific types (pericytes, fibroblasts and vascular smooth muscle cells), and shared organoid-fetal mesenchymal cells. Off-target cell-types were restricted to the organoids, except for *STMN2*+ neuronal cells also found in the week 8 fetal dataset. Fetal- and adult-specific endothelial and immune populations were absent in organoids, consistent with their avascular state. Pairwise correlation (Spearman *ρ*, Supplementary Fig. 6) in expression of data-driven highly variable genes between clusters supported the random forest classification and joint clustering: we found high correlation between nephron cell types and poor correlation of mesenchymal-4 with fetal and adult mesenchymal cell types.

**Fig. 2:**
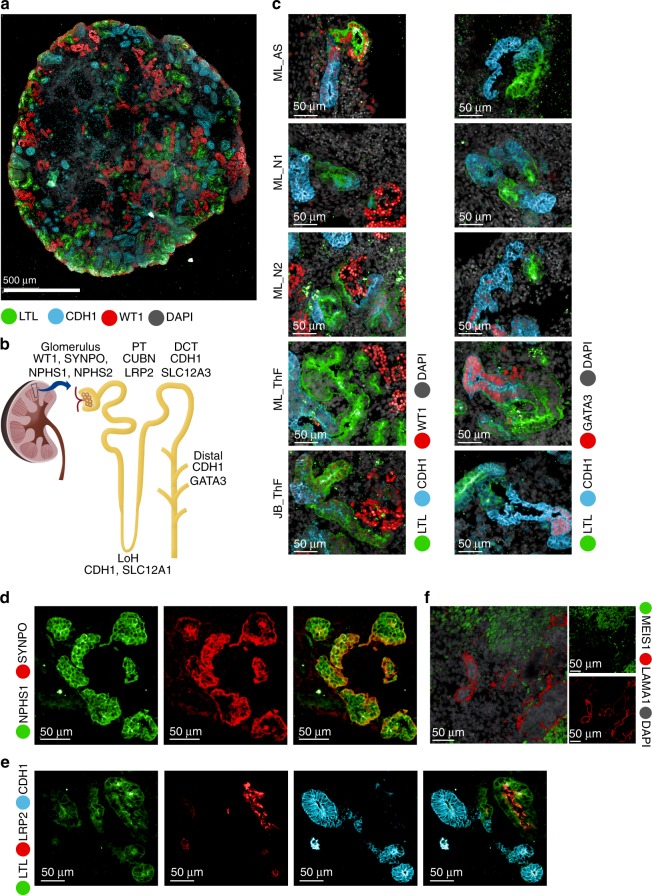
IF validation of markers derived from the single-cell data in mature kidney organoids. **a** IF staining of an entire kidney organoid with segment specific markers as shown. **b** Schematic of kidney nephron with major cell types and canonical markers annotated. **c** Immunofluorescence staining of D29 kidney organoids for podocyte (WT1), proximal tubule (LTL), and distal tubule (CDH1 and GATA3) across two protocols (JB, ML) and four cell lines (AS, N1, N2, ThF). IF staining for validation of markers identified in the single-cell data: **d** NPHS1 colocalized with the podocyte-specific marker SYNPO and **e** LRP2 colocalized with the proximal tubular marker LTL (bottom). **f** IF staining validation for MEIS1-positive mesenchymal cells in D29 organoids. LAMA1 indicates basement membranes. MEIS1 staining of mesenchymal cells appropriately surrounds LAMA1-defined tubular nephron structures.

We validated cell type markers using immunofluorescence (IF) staining (Fig. 2a–c, Supplementary Figs. 7, 8). Markers for podocyte (WT1), proximal tubule (LTL) and distal nephron (CDH1) were detected across all lines and protocols, although expression of GATA3, a marker of the developing ureteric bud (UB), could only be detected in N2 and ThF^7,35^ (Fig. 2c). We also validated the markers used to annotate specific cell clusters: NPHS1 (podocyte cluster), colocalized with the podocyte-specific marker synaptopodin (SYNPO) (Fig. 2d); LRP2 (proximal tubule cluster), colocalized with LTL, a lectin specific to the proximal tubule (Fig. 2e); CDH1 (distal tubular compartment) marked tubules distinctly from proximal LRP2/LTL positive tubular structures (Fig. 2e). We also validated MEIS1, a gene enriched in kidney mesenchymal cells, by IF staining, where it was shown to localize appropriately to the interstitium, defined as the cellular space outside laminin (LAMA1) positive basement membrane structures (Fig. 2f).

### Cell proportion variability is driven by off-target cells

**Fig. 3:**
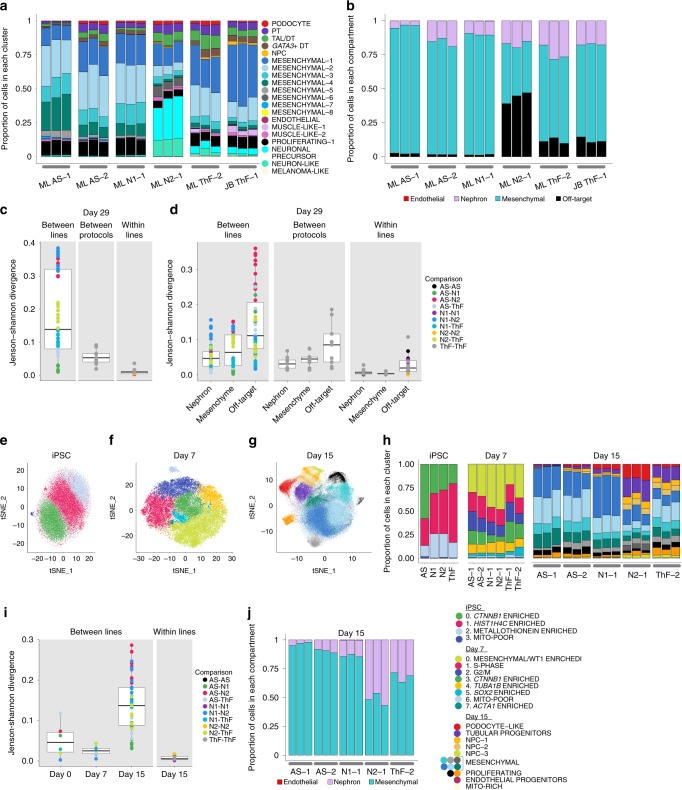
Variability in cell type proportions detected by scRNA-seq evident at D15. **a**, **b** Relative proportions of endothelial, nephron, mesenchymal and off-target cell clusters across all replicates of D29 organoids. Annotations as shown. **c**, **d** Comparison of cell-type composition between D29 organoids as determined by boxplots of the Jenson−Shannon Divergence (JSD) scores. Each point on the plot is a pair-wise (color) measure of JSD between two organoids. Legend indicates annotation for pairs of iPSC lines. The center line of the boxplots indicates the median, and the bottom and top lines of the box indicate the first and third quartiles respectively of the JSD scores. Outliers are indicated as dots beyond the whiskers; whiskers stretch up to +−1.5*IQR on both sides. **c** Organoid compositional differences are greater between lines than between different protocols for the same line or between replicates of the same line and protocol (within lines). **d** Organoid compositional heterogeneity is greatest in the off-target compartment followed by the mesenchyme and the nephron compartment in all three comparison groups (between lines, between protocols and within lines). t-SNE plot of single cells from **e** iPSC, **f** D7, and **g** D15 of the organoid differentiation protocol. **h** Comparison of relative cell type proportion across iPSC lines of cell clusters shown in (**e**−**g**). **i** Compositional differences are greatest between lines at D15 and the least at D7. **j** Compartment-specific cell-type proportions at D15 across all lines and replicates.

Cell type proportions in mature D29 organoids were consistent between replicates of independent clones, but they varied between iPSC lines (Fig. 3a, b). We quantified the differences in cell proportions by computing the Jenson–Shannon Divergence^36^ (JSD is a measure of compositional difference between two frequency vectors with a value between 0 and 1, where smaller values mean more similar) within- and between- iPSC lines (Fig. 3c). Differences between lines (average JSD = 0.18, sd = 0.13) superseded differences between independent clones within the same line (average JSD 0.01, sd 0.01), and between different protocols for the same line (average JSD = 0.06, sd = 0.02) (Fig. 3c). N2 was most different (divergent) from both AS and N1 iPSCs that were the most similar. For example, podocytes were captured across all experimental conditions and at similar proportions to fetal kidney (Fig. 3a and Supplementary Fig. 9A, B), but varied between 1.33% (N1, averaged across replicates) to 2.58% (ThF) between lines on the ML protocol. AS, passage 1 (AS-1) was an outlier, with lower overall nephron numbers (0.3%). The JB protocol captured an average of 0.81% podocytes. Similarly, the distal nephron compartment (including TAL and *GATA3*+ distal nephron cells) ranged in average proportion from 2.34% (N1) to 6.95% (ThF), with an average 1.45-fold-change between protocols. In general, N1 organoids had the lowest average nephron cell proportions, followed by AS, N2 and ThF. *GATA3*+ distal-like cells were more abundant in ThF iPSCs, independent of protocol. *GATA3* expression in AS and N1 was lower than in ThF and N2 (Supplementary Fig. 2), as confirmed by IF (Fig. 2c).

To make higher level comparisons, we looked at four groups: nephron, mesenchymal, off-target and endothelial compartments (Fig. 3b, Supplementary Table 3). The nephron compartment was on average 16.7%, of all cells (9.89% in N1 to 24.1% in ThF). The N1 line had the largest average relative proportion of EC (0.1%), with a global average of 0.05% across all lines. Mesenchymal cells were 82.8% of all cells in AS and 88.6% in N1 organoids, but only 64.2% in ThF and 39.2% in N2. Off-target cells varied markedly by iPSC line, from 1.6% in AS-1 and N1 to 43.7% in N2 (Fig. 3b). To determine variability within each compartment, we again computed the JSD (Fig. 3d). The nephron and mesenchymal compartments were consistent (nephron mean JSD between lines = 0.05, sd = 0.038; mesenchymal mean JSD = 0.07, sd = 0.05), whereas the off-target compartment was variable between lines (mean JSD = 0.14, sd = 0.09) and protocols (Fig. 3d). N2 organoids were most divergent from AS in off-target composition. Notably, the ratio of mesenchymal to nephron cells was inversely related (Spearman correlation *ρ* = −0.84) to the proportion of off-target cells. Higher proportion of off-targets (N2, ThF: 11.7%, JB ThF: 12.2%) resulted in lower mesenchymal:nephron ratios (N2: 2.29, ThF: 2.66, JB ThF: 4.05) and vice-versa (off-targets—N1, AS:1.59%; mesenchymal:nephron—N1: 8.96, AS: 5.31). In contrast, the mesenchyme proportions were lower in both adult and fetal kidney, both overall (20.3% in adult, 19.7% in fetal week 17, Supplementary Fig. 9C) and relative to nephron cells (1.23 in adult, 0.47 in fetal). In summary, we noticed greater organoid heterogeneity between iPSC lines than between replicates within a line, or between protocols, with the off-target compartment contributing the most variability.

### Variability in cell type proportions emerges by D15

To explore whether the iPSC-line associated differences were associated with their basal state^19,37^ at D0 or the process of differentiation, we compared the single-cell profiles collected at D0, D7, and D15 for each organoid culture.

Analysis of 42,433 single cells from the four undifferentiated iPSC lines at D0 (Fig. 3e, h and Supplementary Fig. 10A, Supplementary Data 4) showed even distribution across identified cell clusters, indicating uniformity across initial iPSC lines. To determine if there were clusters of cells primed towards a particular developmental germ layer, we scored organoids using known transcriptional signatures for the three germ layers (Supplementary Data 5). We did not observe subclusters with signatures for any specific germ layer^38^, or primed for differentiation^39^ (Supplementary Fig. 11A). The four cell clusters had representation from each of the iPSC lines (avg JSD = 0.05, sd = 0.04, Fig. 3h, i), and from all cell-cycle phases (Supplementary Fig. 11B).

Similarly, at D7, developing organoids from all iPSC lines expressed appropriate markers of mesodermal differentiation with actively proliferating cells^21^ (Supplementary Fig. 11C, D; Supplementary Data 6) and little variability between lines (Fig. 3f, h). Differences in proportions were small (avg JSD = 0.02, sd = 0.01, Fig. 3i). Notably, clusters 3, 5 and 0 appropriately expressed *CTNNB1* and *SOX11*, *SOX2*, and *WT1*, respectively, genes upregulated during the differentiation of intermediate mesoderm (IM) into the metanephric mesenchyme (MM) or the UB^40,41^ (Supplementary Fig. 10B). We did not observe nephron cell types in D7 organoids.

At D15, organoids had multiple subsets of NPCs and a distinct population of epithelial cells broadly expressing tubular progenitor markers (PAX2, LHX1; Supplementary Fig. 12A, Supplementary Data 7). Across all iPSC lines, we observed early patterning of the nephron (reflected by expression of proximal (*CUBN*) and distal (*MAL*, *WFDC2*, *POU3F3*) markers; Supplementary Fig. 8A), proliferating cell populations (e.g., cells in the Mesenchymal-4 cluster expressed *CTNNB1*, *PTPRS*, *CDC42*, and *SOX11*, similar to *CTNNB1*-expressing cells in clusters in the iPSCs and D7 organoids; Supplementary Fig. 12A), and a distinct subset of podocyte-like cells (Fig. 3g, h; Supplementary Fig. 12A). In contrast to D7 and D0 (Fig. 3i), there were notable differences in composition between iPSC lines (avg JSD = 0.14, sd = 0.07; Fig. 3g, h, i). The proportion of nephron-like cells ranged from 0.08% in AS to 56.9% in N2 D15 organoids (Fig. 3h, j). Importantly, we also noted heterogeneity in endothelial progenitors (consistent with D29 organoids): N1 had the highest average relative proportions (0.32% vs. 0.05% among other lines; Fig. 3j).

Although no distinct off-target cells were found at D15, N2 and ThF organoids had a small population of cells expressing the neuronal progenitor *SOX2* within the mesenchymal compartment (Supplementary Fig. 12B). The mesenchyme:nephron ratios were also lower in N2 and ThF D15 organoids (Fig. 3j). Hence, organoids with higher nephron proportions at D15 had a distinct pool of *SOX2*+ cells, and went on to develop a higher proportion of off-target cells at D29, associated in turn with lower D29 mesenchymal to nephron ratios (Fig. 3b, j). Further, we performed immunofluorescence staining for SOX2 on D15 organoid sections from all lines (Supplementary Fig. 12C). We found that SOX2 protein was detectable in ThF and N2 organoids (which have more off target cells at D29, Fig. 3b) but negligible in AS and N1 (which have few off target cells at D29, Fig. 3b). We thus concluded that SOX2 expression at D15 may be a source of variability in organoid differentiation. Finally, we performed joint clustering analysis of D0, D7 and D15 organoids (Supplementary Fig. 13, Supplementary Data 8) from the ThF line. We found that: (1) D0 had clusters with specific expression of markers of pluripotency (e.g. *POU5F1, L1TD1*); (2) D7 had clusters with high expression of mesodermal markers (e.g. *DKL1, HOXB9, HOXD1, HOXC8*); (3) D15 had proximal and distal nephron progenitor clusters (e.g. proximal: *WT1, FOXC2, NOTCH2*, distal: *IRX3, JAG1, PAX2, PAX8, LHX1*); (4) proliferating states were shared (e.g. *UBE2C*, *TOP2A*, *MKI67, CENPF*). Taken together, off-target populations, detected by D15, were the most variable between organoids.

### Concordant expression of developmental programs across organoids

**Fig. 4:**
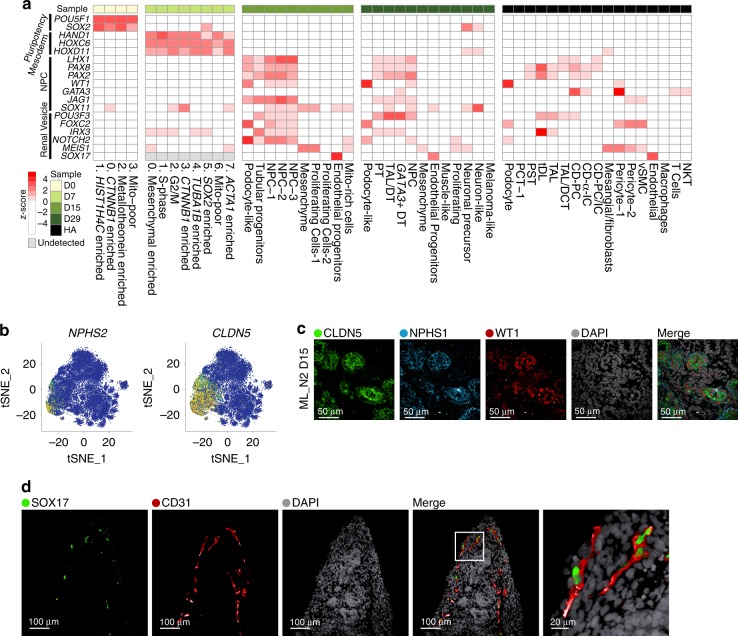
Concordant expression of developmental programs across organoids from four human iPSC lines. **a** Heatmap of expression patterns for major nephrogenesis markers across organoid differentiation time points (iPSC D0, D7, D15, and D29, averaged across four cell lines, ML protocol) and human adult kidney. Expression values were row-normalized to obtain *z*-scores; red color indicates positive *z*-scores. **b** Canonical (*NPHS2*) and data-derived (*CLDN5*) podocyte marker genes superimposed in tSNE plots from D15 organoids (N2 line, ML protocol). **c** IF staining of D15 kidney organoid (N2 line, ML protocol) for CLDN5 as a marker of early podocyte differentiation derived from the single-cell data. Additional canonical podocyte markers (NPHS1, WT1) and DAPI staining as shown. **d** IF staining of D29 kidney organoid (AS line, ML protocol) for SOX17 and CD31, markers of endothelial cells.

Next, we tested whether known transcription factors (TFs) and critical genes involved in nephrogenesis^40,41^ (Supplementary Table 4) are appropriately expressed in the respective clusters in organoids from four different iPSC lines, compared to adult kidney. Overall, key developmental programs were expressed at expected time points and transitions, in a comparable manner across different iPSC-derived organoids (Fig. 4a, Supplementary Fig. 14,15A). First, core pluripotency TFs were expressed during the iPSC stage and decreased subsequently (D7-D29). Notably, *SOX2*, a neuronal progenitor marker^42^, re-appeared in D29 organoids in off-target neuron cells (Fig. 4a). Next, at D7, prior to the CHIR pulse (Fig. 1a), cells across all clusters had high expression of markers of mesoderm development, such as *HAND1*^43^ and *HOX11* genes^44^. With organoid self-assembly between D7 and D15, IM inducing genes from D7 were downregulated in D15, while nephron progenitor genes were upregulated in a nephron-specific pattern: *JAG1*, *LHX1*, *PAX2*, *PAX8* in the epithelial clusters and *WT1* in the nephron progenitor population and podocyte-like cells (Fig. 4a). At D15 we first observed a *SOX17*-positive endothelial progenitor cell population that persisted in D29 (Fig. 4a, Supplementary Fig. 9C). Nephron patterning became more evident at D29: *FOXC2* and *WT1* were prominent in podocytes, whereas *IRX3* and *POU3F3* had a distinct distal pattern (Fig. 4a, Supplementary Fig. 15A).

Of several data-derived markers enriched in the podocyte population at D15 (*CTGF*, *CLDN5*, *SOST*, *SPARC*; Fig. 4b, Supplementary Fig. 15B), we validated CLDN5 (claudin 5, an integral membrane protein controlling tight junctions^45^ in many cells including podocytes^46^) in nephrin (NPHS1) and WT1-positive podocytes of D15 organoids (Fig. 4c). To validate the endothelial precursors identified in our analysis of D15 organoids, we stained for SOX17 (localized to EC nuclei) and showed that it colocalized with CD31, a classical endothelial marker (Fig. 4d).

### Compartment-specific expression of disease-associated genes

**Fig. 5:**
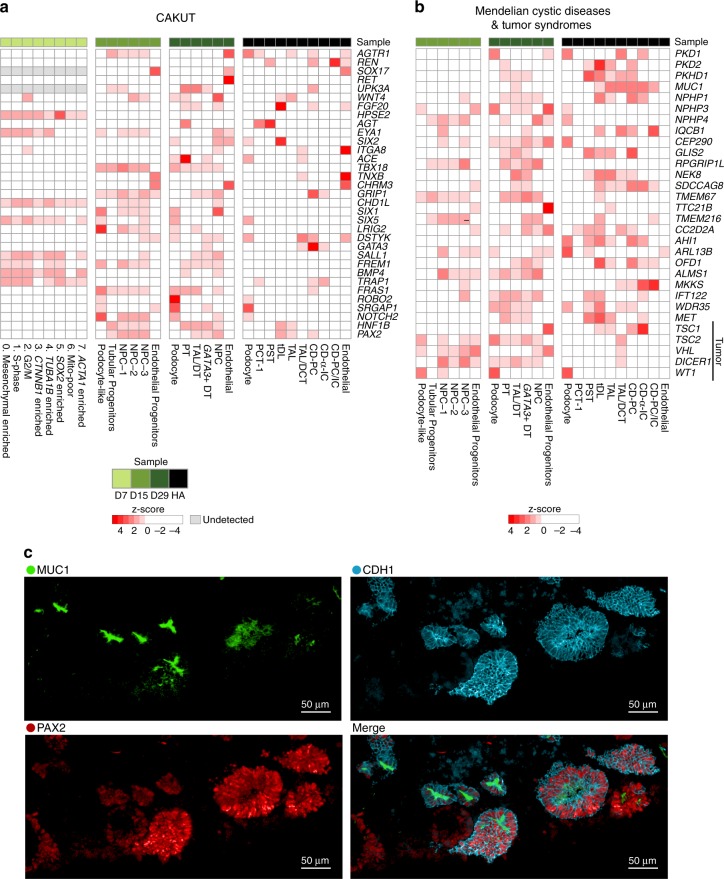
Genes associated with kidney diseases are expressed in expected compartments across organoids from four human iPSC lines. Heatmap of cell-type-specific gene expression (ThF line, ML protocol) in organoids and human adult kidney. Genes implicated in monogenic causes of **a** congenital abnormalities of the kidney and urinary tract (CAKUT), and **b** mendelian renal cystic diseases and tumor syndromes. **c** IF staining validates the expression of MUC1 and PAX2 in the distal tubule in D29 kidney organoids. CDH1 serves a distal tubular marker. Note appropriate apical expression of MUC1 in distal tubular epithelial cells.

To assess the applicability of kidney organoids from different iPSC lines for the study of genetic kidney diseases^5,11,13^, we determined the expression of genes causing congenital anomalies of the kidney and urinary tract (CAKUT), hereditary renal cystic (HRC) diseases and hereditary tumor syndromes (Fig. 5a, b, Supplementary Figs. 16, 17; Supplementary Data 9), as well as genes from Genome Wide Association Studies (GWAS) of chronic kidney disease (CKD)^47^ (Supplementary Fig. 18) and mendelian glomerular diseases^48^ (Supplementary Fig. 19).

Organoids from all lines reproducibly expressed genes associated with progressive kidney diseases (including adult onset diseases) in at least one compartment. Genes associated with embryonic and early childhood abnormalities (CAKUT and HRC) were broadly expressed in developing organoids from all iPSC lines as early as D7 (i.e. *CHD1L*^49^ at D7). Appropriately, developmental genes enriched in organoids were absent in adult kidney (i.e. *RET*, *HPSE2*). Some genes achieved high levels of expression and cell-type specificity in mature organoids, in a pattern similar to adult kidney. For example, *PTPRO* (from CKD GWAS and monogenic glomerular diseases) was appropriately expressed in D29 and adult human podocytes^50^ (Supplementary Figs. 18, 19). Similarly, *PAX2* and *MUC1* (from CAKUT and cystic diseases, respectively) were highly expressed in D29 distal tubules, similar to their human adult expression pattern^51^ (Fig. 5a, b; Supplementary Figs. 16, 17). We validated these markers derived from scRNA-seq analysis by IF staining, which confirmed the coexpression of PAX2 and MUC1 in CDH1+ distal tubular epithelial cells in mature organoid sections (Fig. 5c). In summary, kidney organoids from four different iPSC lines could serve as reasonably faithful surrogates of human kidney tissue for the study of a broad array of kidney diseases.

### Organoid transplantation in mice diminishes off-target cells

**Fig. 6:**
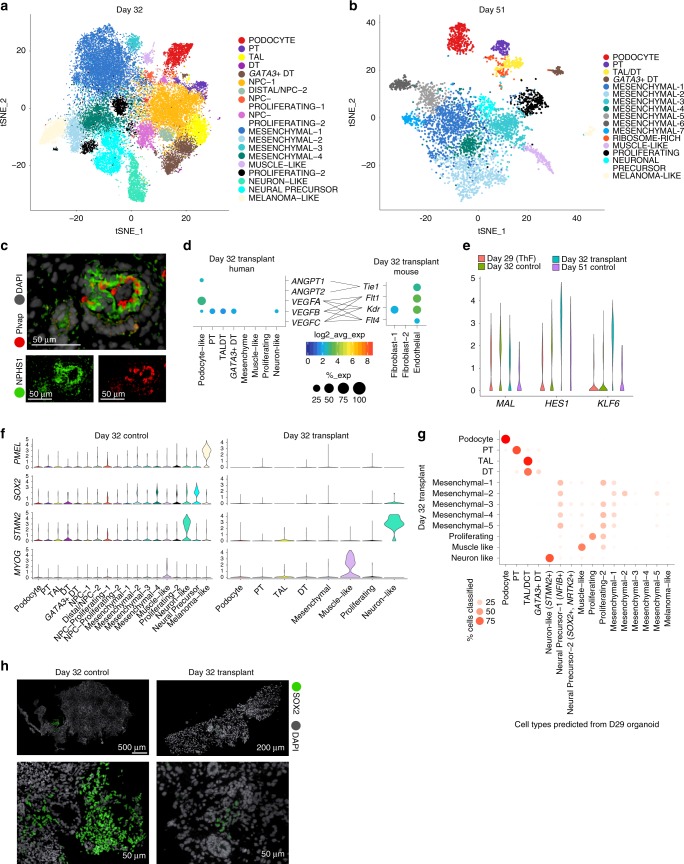
Transplantation of human organoids into mouse diminishes off-target cells. t-SNE plots of organoids in prolonged in vitro culture reveal cell clusters in **a** D32 and **b** D51 organoids similar to clusters from D29 organoids. **c** Transplanted and vascularized organoid at D51. IF staining shows human podocytes (anti-human NPHS1 antibody) at the outer perimeter and mouse endothelial cells (anti-mouse Plvap antibody) lining the internal perimeter of a glomerular structure. Gray, human and mouse nuclei (DAPI). **d** Dotplots indicating ligand and receptor pairs involved in putative cross-talk between human podocytes and mouse endothelial cells of transplanted organoids. **e** Violin plots of expression levels for *KLF6* and *HES1* in the distal *MAL*+ cluster from D29, D32 and D51 organoids grown in vitro, compared to D32 transplanted organoids. **f** Violin plots demonstrate that *PMEL*+ melanoma cells and *SOX2*+ neuronal cells were diminished after transplantation. *MYOG*+ muscle cells and *STMN2*+ neuron-like cells persisted. **g** Random Forest Classifier shows the relation of cell clusters in transplant organoids compared to organoids grown in vitro. Melanoma and *SOX2*+ neuronal precursor cells are not detected in D32 transplant organoids. **h** IF staining validation for SOX2 (green) in D32 control organoids compared to diminished abundance in D32 transplanted organoids. Human and mouse nuclei (DAPI, gray).

To improve overall organoid quality, we sought ways to reduce off-target cells. First, we determined that prolonged organoid culture in vitro, up to 51 days, did not reduce the proportion of off-target cells, as assessed by scRNA-seq of organoids grown in vitro at day 32 (D32) and day 51 (D51) (Fig. 6a, b; Supplementary Fig. 20). D32 and D51 organoids had largely similar cell populations as in D29 organoids (Supplementary Fig. 21, Supplementary Data 10), including muscle, neuronal and melanoma off-target cell clusters. In particular, we noted two clusters of neuronal off-target cells at D29, D32 and D51: *STMN2*+ neuron-like cells (D29, D32) and *SOX2*+ *NTRK2*+ neuronal-precursor cells (D29, D32, D51; Supplementary Fig. 2, 20B).

Kidney development requires signaling from adjoining vasculature^52^, but EC were minimal in organoids grown in vitro (Figs. 1c and 3a). Recently modified protocols emphasizing shorter exposure to CHIR^15^ or exogenous addition of VEGF^14^ increased the EC population in vitro, but these EC did not invade the glomerular capsule^14,15^. Organoid transplantation under the kidney capsule of immunodeficient mice allows mouse EC to infiltrate the transplanted kidney organoid and promote vascularization^15,53–55^. However, to date, transplanted organoids have not been characterized at single-cell resolution.

We transplanted D18 and D25 organoids (ThF line) under the mouse kidney capsule of immunodeficient mice, recovered D18 transplants at 14 (D32 organoid transplants) and D25 transplants at 26 days following transplantation (D51), and profiled them by scRNA-seq, along with D32 control organoids grown in vitro (Supplementary Fig. 22A-D). A lower yield of single cells was obtained from the D51 organoid transplants due to technical reasons. We distinguished the human from mouse cells by read alignment to the combined genome reference (Methods) and confirmed that the human nephron cells in D32 transplants (Supplementary Fig. 22 D,E; Supplementary Data 11) corresponded to D29 nephron clusters. We confirmed that transplanted human organoids at D51 were vascularized by mouse EC^56^: IF showed mouse Plvap-positive EC apposed to human podocytes (NPHS1) (Fig. 6c, Supplementary Fig. 22C). A few human nuclei were present within the mouse parenchyma, suggesting a modest reciprocal invasion of human cells into mouse tissue (Supplementary Fig. 22C).

To gain further mechanistic insights at the molecular level, we performed receptor-ligand analysis. This showed *VEGFA* and *ANGPT1* most highly expressed in podocytes (Fig. 6d) and correspondingly, the VEGF receptors *Flt1*, *Kdr* and *Flt4*^57–59^ and the angiopoietin receptor *Tie1* most highly expressed in mouse *Plvap*+ EC (Fig. 6d), suggesting that these cell types could interact. Further, tubular epithelial cells from transplanted organoids had increased expression of *KLF6*, a TF involved in the development of the UB and the CD^60^, and the Notch effector *HES1*, which plays a role in proximal to distal patterning in kidney nephrogenesis^61^, in *MAL*+ distal tubular cells (Fig. 6e). These upregulated gene programs suggested enhanced maturity states in distal tubular cells from D32 transplanted organoids compared to in vitro D32, D29, or D51 controls.

Strikingly, we found little off-target expression of *SOX2* (neuronal precursor cells) or *PMEL* (melanoma cells) in D32 transplanted organoids compared to controls, suggesting that transplantation diminished off-target cells (Fig. 6f; Supplementary Fig. 23A). *MYOG*-positive muscle cells persisted in D32 transplanted organoids (Fig. 6f, Supplementary Fig. 23A). We also noted that a rare *STMN2*+ neuronal cluster persisted in D32 transplants. This cluster was uniquely and highly correlated with the neuronal cluster in week 17 fetal kidney (Spearman *ρ* = 0.82; Fig. 1f, Supplementary Fig. 23B), suggesting these *STMN2+* cells are not true off-target cells. A set of enriched genes (*GAL*, *CHGA*, *CHGB*; Supplementary Fig. 24) was shared between this cluster in human fetal kidney and D32 transplanted organoids (Supplementary Fig. 24A). *CHGA* and *CHGB* were also detectable in a small number of cells within the *STMN2*+ cluster in D29 control organoids (Supplementary Fig. 24B), suggesting that transplantation selected for and promoted this on-target *STMN2*+/*CHGA*+/*CHGB*+ cluster. We applied a classifier to further assess the relation of all cell clusters in transplanted organoids to organoids grown in vitro (Fig. 6g). Consistently, no transplanted organoid cells corresponded to *SOX2*+ *NTRK2*+ neural precursors or *PMEL*+ melanoma-like cells (Fig. 6g). In line with these findings, IF staining showed diminished SOX2 staining in D32 transplanted organoids compared to numerous SOX2-positive cells in controls (Fig. 6h). We thus concluded that transplantation abrogated *SOX2+* neuronal and *PMEL+* melanoma off-target cells, and may have promoted organoid maturation toward a more advanced human-like state. Taken together, these data showed that transplantation reduced off-target cells, improving organoid quality.

## Discussion

In this study, we present a comprehensive census of human kidney organoids in comparison to human adult and fetal kidneys at single-cell resolution spanning multiple iPSC cell lines, time points, protocols, and replicates including transplantation into mice, in a total of 450,118 cells. This analysis provided answers to several critical questions regarding organoid reproducibility, faithfulness and quality.

First, we addressed a critical need in the field by comparing kidney organoids derived from four human iPSC lines at single-cell resolution. The large number of cells and replicates sequenced across different conditions in this study afforded us the opportunity to retrieve numerous cell types, and perform robust statistics such as the Jenson–Shannon Divergence test^36^ to gain quantitative insights into organoid reproducibility. By comparison with adult and fetal human kidneys, major nephron cell types were identified in D29 organoids from all four iPSC lines, in support of the robustness of current protocols. Podocytes and PT cells were well developed, whereas distal tubular cells were less differentiated. At single-cell resolution, we also found that the iPSCs themselves had comparable pluripotency (at D0) and modest variability at D7. However, D15 organoids showed greater variability in cell proportions and a distinct pool of *SOX2*+ cells, suggesting that variability in mature D29 organoids is likely derived from off-target programs at a point in organoid development between D7 and D15. The pattern of variability in cell composition was then maintained through D29, driven by the off-target compartment. Importantly, organoids with higher proportions of NPC at D15 went on to develop more off-target cells at D29. Hence, our data reveal that future improvements in organoid protocols should focus on the time interval between D7 and D15, aiming to reduce the persistence of progenitor cells that drive the development of off-target cell populations.

Second, the applicability of organoids as a tool for discovery was bolstered by our data showing that kidney organoids from four different iPSC lines could serve as reasonably faithful surrogates of human kidney tissue for the study of a broad array of kidney diseases. For example, we validated the expression of *MUC1* in the distal tubular compartment of D29 organoids. Mutations in *MUC1* are a cause of autosomal dominant tubulointerstitial kidney disease^62,63^, a rare kidney disease without a cure. Efforts to find cures for genetically defined diseases may greatly benefit from our ability to study their mechanisms in patient iPSC-derived organoids^64^. This study provides an important foundational step in this direction; our work suggests that organoids derived from individual patients can indeed be used as a tool to fuel biological discovery and therapeutics. This study may thus serve as a valuable reference when trying to make meaningful comparisons between iPSC-derived kidney organoids from different patients.

Finally, we addressed the critical issue of organoid quality with respect to off-target cells. Recent reports focused on eliminating neuronal off-target cells from kidney organoids by using an NTRK2 blocker^17^. However, *NTRK2* is not only expressed in off-target neuronal cells, but also abundantly expressed in podocytes^65,66^ in both fetal human kidneys^67^ and developing organoids (Supplementary Fig. 24A, C), raising the concern that NTRK2 blockers applied to organoid cultures may adversely affect podocyte differentiation and function. Here we identified organoid transplantation as an alternate approach to diminish off-target cells. Transplantation under the mouse kidney capsule diminished *SOX2*+ neuronal precursors and *PMEL*+ melanoma cells. In addition, we uncovered an on-target *STMN2*+/*CHGA*+/*CHGB*+ cluster that was selected and promoted in transplanted organoids, suggesting that the elimination of off-target cells may also benefit organoid maturity. Future studies will be required to test if earlier organoid transplantation, as early as D7, may eliminate off-target cells and enhance organoid formation, especially UB and CD development (building on gene programs such as *KLF6* and *HES1* that we found uniquely upregulated in transplanted organoids). The advantages of transplantation are also supported by recent studies with modified protocols^14,15^ that, on the one hand, achieved an increase in the EC population in vitro, but on the other hand found that the EC did not invade the glomerular capsule^14,15^ unless they were transplanted in vivo^15^. Therefore, as it stands, organoid transplantation is a reliable method for vascularization and the removal of off-target cells. While the low throughput of this method makes it impractical for initial drug screening purposes (where in vitro systems^14^ may be best), we speculate that future studies of promising lead compounds in transplanted, vascularized organoids will provide valuable data about compound efficacy, its metabolism by human kidney cells, and kidney-specific toxicities.

In conclusion, our study yields deep insights into iPSC-derived kidney organoids, illuminating their reproducibility, pinpointing the source of variability, and showing that transplantation reduces off-target cells. This comprehensive census, enabled by scRNA-seq technology, should serve as a foundational resource for the community, fueling the development of much-needed therapies for patients with kidney diseases.

## Methods

### Cell culture and chemicals

mTeSR1 (Stem Cell Technologies, no. 85870), Gentle Cell Dissociation Reagent (Stem Cell Technologies, no. 7174), ROCK Inhibitor Y-276*32* (Stem Cell Technologies, no. 72304*)*, STEMdiff^TM^ APEL^TM^2 (Stem Cell Technologies, no. 05270), Accutase (Stem Cell Technologies, no. 07920), Corning Matrigel (Stem Cell Technologies, no. 354277), six-well transwell plate (Stem Cell Technologies, no. 3450), CHIR99021 (R&D Systems, no. 4423/10), Activin A (R&D Systems, no. 338-AC), Human recombinant FGF-9 (Peprotech, no. 100-23), NOGGIN (Peprotech, 120-10C), Heparin (Sigma-Aldrich, no. H4784-250mg), Phalloidin-647 (ThermoFisher, no. A22287, 10 U/mL in 1.5% Tween-20), DAPI (ThermoFisher, no. 62248, 1: 10,000 in PBS) and ProLong^TM^ Gold antifade reagent (ThermoFisher, no. P36930).

### Antibodies and immunofluorescence

WT1 (ThermoFisher, no. PA5-16879, 1:100), ECAD, SYNPO (Abcam, no. ab117702, 1:200), MUC1 (Abcam, no. ab80952, 1:500), fluorescein-labeled LTL (Vector Laboratories, no. FL-1321, 1:300), GATA3, SOX2 (Cell Signaling Technology, no. 5852, 3579 1:300), Laminin (Sigma-Aldrich, no. L9393, 1:500), MEIS1 (Activemotif, no. ATM39795, 1:300) CD31 (BD Pharmingen, no. 555444, 1:300), SOX17 (R&D Systems, no. AF1924, 1:300), LRP2 (Santa Cruz Biotechnology, no. 515772, 1:100), PAX2 (Zymed laboratories, no. 71-6000, 1:300). All donkey Alexa Fluor secondary antibodies were purchased from Invitrogen (1:1000). Human nuclei (antibodies online, no. ABIN361360, 1:300), MECA-32 (BD Biosciences, no. 553849, 1:300), NPHS1 (R&D Systems, no. AF4269, 1:300), Claudin-5 Antibody (Novus Biologicals, no. NBP2-66783, 1:300) and NTRK2 (abclonal A2099, 1:300). All the secondary antibodies were purchased from Thermo Fisher Scientific. Donkey anti-Rat Alexa Fluor 488 (Cat# A-21208, 1:1000), donkey anti-Sheep IgG Alexa Fluor 568. (Cat# A-21099, 1:1000), donkey anti-rabbit IgG Alexa Fluor 568 (Cat# A10042, 1:1000), donkey anti-mouse IgG (H + L) highly cross-adsorbed secondary antibody, Alexa Fluor 568 (Cat# A10037, 1:1000), goat anti-mouse IgM Alexa Fluor 488 (Cat# A-21042, 1:1000), goat anti-rabbit IgG (Alexa Fluor 405 (Cat# A-31556, 1:1000), donkey anti-rat IgG Alexa Fluor 488 (Cat# A-21208, 1:1000) and goat anti-rat IgG Alexa Fluor 647 (Cat# A-21247, 1:1000).

### iPSC culture

Human Episomal iPSC Line (ThermoFisher, no. A18945, ALSTEM, no. iPS16). N1 line (S1930 CB A) N2 line (S1973 WR I) were derived from erythroblasts using CTS™ CytoTune™-iPS 2.1 Sendai Reprogramming Kit (ThermoFisher, no. A34546) at Harvard Stem Cell Institute (HSCI) iPS Core Facility. Normal iPSC lines (N1 and N2) were generated with informed consent under Broad ORSP-3414. The N1 and N2 cell lines were characterized for pluripotency and spontaneous differentiation to the three germ layers using qPCR based on standard protocols at the HSCI Core Facility. All iPSC cultures were maintained in mTeSR1 medium in T25 flasks coated with Matrigel. Cells were passaged using Gentle Cell Dissociation Reagent. All lines were confirmed to be karyotype normal and maintained below passage 15 and all the cell lines were routinely checked and were negative for mycoplasma.

### Kidney organoids differentiation from iPSCs

ML and JB kidney organoid differentiation protocols were adopted from the methods^7,8^ with slight modifications. ML protocol: 375K iPS cells were plated in a T25 flask in mTeSR1 media and ROCK Inhibitor, Y-27632 (10 μM). After 24 h, cells were treated with CHIR99021 (8 μM) in APEL2 medium for 4 days, followed by recombinant human FGF-9 (200 ng/mL) and heparin (1 μg/mL) for an additional 3 days. At day 7, cells were dissociated into single cells using ACCUTASE^TM^. 500 K cells were pelleted at 350 × *g* for 2 min (twice with 180° flip) and transferred onto a six-well transwell membrane. Pellets were incubated with CHIR99021 (5 μM) in APEL2 medium for 1 h at 37 °C. After, the medium was changed to APEL2 supplemented with FGF-9 (200 ng/mL) and heparin (1 μg/mL) for an additional 5 days, and an additional 2 days with heparin (1 μg/mL). The organoids were maintained in APEL2 medium with no additional factors until days 29–51 for downstream experiments. Medium was changed every other day.

*JB protocol*: iPS cells were plated as described above. After 24 h, cells were treated with CHIR99021 (10 μM) and NOGGIN (5 ng/mL) in APEL2 medium for 4 days, followed by Activin A (10 ng/mL) for 2 days, and FGF-9 (10 ng/mL) for an additional 2 days. At day 8, the cells were dissociated and transferred to a six-well transwell plate as described above. Pellets were incubated with CHIR99021 (3 μM) and FGF-9 (10 ng/mL) in APEL2 for 2 days, followed by 4 days with only FGF-9 (10 ng/mL). Organoids were maintained in APEL2 medium with no additional factors until harvest at days 25–28 for downstream experiments. Medium was changed every other day.

### Single-cell isolation for 10X genomics

Day 28 mature kidney organoids were washed twice with PBS and incubated with Accumax (Stem Cell Technologies, no. 07921) for 10 min at 37 °C and were dissociated into single cells using a 27G syringe (BD Biosciences, no. 305540). Cells were spun down at 350 × *g* for 5 min, resuspended in PBS, filtered through a 40 µm filter (Corning, no. 352340), and checked for viability.

### Library preparation and single-cell sequencing

Single cells were processed through the 10X Chromium 3′ Single Cell Platform using the Chromium Single Cell 3′ Library, Gel Bead and Chip Kits (10X Genomics, Pleasanton, CA), following the manufacturer’s protocol. Briefly, 10,000 cells were added to each channel of a chip to be partitioned into gel beads in emulsion (GEMs) in the Chromium instrument, followed by cell lysis and barcoded reverse transcription of RNA in the droplets. Breaking of the emulsion was followed by amplification, fragmentation and addition of adapter and sample index. Libraries were pooled together and sequenced on Illumina HiSeq.

### Immunofluorescence

Organoids were fixed in 4% paraformaldehyde (Alfa Aesar, no. J61899-AP), cryoprotected in 30% sucrose solution overnight, embedded in optimum cutting temperature (OCT) compound (VWR, no. 25608-930), flash frozen in dry ice with ethanol, and kept at −80^o^C overnight. Organoids were cryosectioned (Leica CM1950 Clinical Cryostat) at 6 µm and mounted on Micro Slides, Superfrost^TM^ Plus (VWR, 48311-703). Slides were washed with PBS (one time, 5 min), blocked for 20 min (5% normal donkey serum, 1.5% Tween-20), and incubated overnight at 4 °C with primary antibody (in blocking buffer). Later the slides were washed with PBS (three times, 10 min each) and incubated with secondary antibody (PBS, 1.5% Tween-20) for 2 h at room temperature. The slides were then washed with PBS (one time, 10 min). For phalloidin staining, the slides were incubated with Phalloidin-647 for 20 min at room temperature and washed with PBS (one time, 10 min). The sections were stained with DAPI for 5 min, washed with PBS (three times, 10 min), and mounted using ProLong^TM^ Gold antifade reagent. Images were obtained by confocal microscopy (PerkinElmer Opera Phenix High Content Screening System).

### Animal experiments

Renal subcapsular transplantation of iPS-derived kidney organoids protocol was adopted from the method^54^. Animal experiments were done at Custom contract research company Biomere (Biomedical Research Model company (https://biomere.com). Biomere has all the IACUC approval for animal experiments and all the animals were housed singly in a conventional housing setting. Recipient mice (*n* = 8, NOD scid gamma (NSG) 8-week-old female mice, The Jackson Laboratory, no. Jax # 005557). Mice were anesthetized with ketamine/dexdomitor and for pain relief animals were injected with buprenorphine. Left kidney was exteriorized and a small insertion was made in the renal capsule. ThF kidney organoids generated using ML protocol (days 7 + 11 and days 7 + 18) were bisected and transplanted onto the left kidney capsule of mice using 24 Ghz catheter. Days 7 + 11 organoids were collected after 14 days of implantation and days 7 + 18 organoids were collected after 26 days of implantation for single-cell RNA sequencing and immunohistochemistry analysis.

### Single-cell isolation from human kidney tissue

Samples of macroscopically normal cortex were obtained from a tumor nephrectomy specimen, distant from the tumor site and after appropriate patient consent, in accordance with IRB and institutional guidelines. Tissue was cut into 1 mm × 1 mm cubes and placed in 0.25 mg/mL liberase TH (Sigma-Aldrich no: 5401135001) dissociation medium. Following further dissection, the tissue was incubated at 37 °C for 1 h in a thermomixer at 600 rpm. Samples were regularly triturated during the incubation period using a 1 mL pipette, after which 10% heat-inactivated FBS RPMI was added to stop the digestion. Centrifugation at 500 × *g* for 5 min at room temperature and removal of the supernatant was followed by the addition of ACK lysing buffer to remove erythrocytes (Thermo Fisher Scientific, no: A1049201). Repeat addition of ACK lysing buffer was performed given the kidney was not perfused prior to removal. Following centrifugation, the resulting cell pellet was incubated with Accumax at 37 °C for 3 min (Stem Cell Technologies, no. 07921). Ten percent FBS RPMI was again used to neutralize the accumax and centrifugation was followed by resuspension of the cell pellet with 0.4% BSA/PBS. The single-cell suspension was then filtered using a 30 μm CellTrics filter (Sysmex America Inc, no: 04-004-2324) with the resulting cell concentration and viability determined using trypan blue. 10,000 cells were then loaded into the 10x Genomics microfluidic system according to the manufacturer’s guidelines (10x Genomics, Pleasanton, USA).

### Study design

Replicates from both sexes were incorporated wherever possible. Organoids were pooled on lanes using a randomized design to ensure that organoids replicates from an individual batch (donor, replicate, condition) were distributed across lanes.

### Preprocessing of 10× droplet-based sequencing outputs

We used the *Cellranger* toolkit (v2.1.1) to perform de-multiplexing using the “cellranger mkfastq” command, and the “cellranger count” command for alignment to the human transcriptome, cell barcode partitioning, collapsing unique-molecular identifier (UMI) to transcripts, and gene-level quantification.

### scRNAseq quality control

We filtered cells to only include barcodes with minimum mapped UMIs of 1000, summarizing to at least 200 genes for downstream analysis. Further, the percentage of reads mapping to mitochondrial genes was capped at 20%.

### Inferring cell types from individual donor organoids and fetal and adult kidney cells

We used the default settings in the *Seurat* R package^68^ (v2.3) for normalization (NormalizeData) of the gene expression counts and identifying variable genes (FindVariableGenes). Briefly for normalization, UMI counts in each gene in a cell are divided by the total UMI count for the cell, divided by a factor of 100,000 and log-transformed to obtain log (TPX + 1) values. After mean-centering and scaling, we performed dimensionality reduction using principal component analyses (RunPCA) on the highly variable genes computed in the previous step. We retained 50 PCs for unbiased clustering (FindClusters) by a shared-nearest neighbor (SNN) graph-based optimization and smart-moving local community detection algorithm. For iPSCs, the first eight principal components sufficiently captured all the variance. The resolution parameter was adjusted as needed: for the human adult sample, resolution of three recovered expected renal cell compartments, for days 28 and 15, resolution of 1 was used. We computed an embedding of the data in 2D space using t-distributed stochastic neighbor embedding (tSNE) in the PC space for visualization (RunTSNE), independent of the clustering step.

### Assignment of cell-identity and gene-set signatures

Cluster-enriched or marker genes were computed using the Wilcoxon-Rank sum test (FindAllMarkers) for differential expression of genes in the cluster cells vs. all other cells, and selecting those genes that pass the adjusted *p* value (Benjamini–Hochberg^69^ FDR) cutoff of 0.05 as cluster-representative. Cluster identity was assigned by comparing data-driven genes with a list of literature-curated genes for kidney developmental and mature cell types provided in Supplementary Table 1. Subclustering was performed when a single cluster represented marker genes from multiple renal epithelial cell types. We checked that cluster membership was not exclusive to a single replicate. Cells were scored for embryonic germ layer-specific gene signatures using Seurat’s “AddModuleScore” function. Seurat’s “CellCycleScoring” function was used to score cells for different stages of cell cycles.

### Curation of gene signatures

The cell-cycle signatures were obtained from Tirosh et al.^70^ (phase-specific genes, aad0501_Table_S5.xlsx) and provided in Supplementary Data 2. The germ-layer signatures were obtained from Tsankov et al.^38^ (nbt.3387-S5.xlsx). Only those genes were included that had weight_2D atleast 0.5 and are provided in Supplementary Data 5. We curated a set of genes, including transcription factors, critical for early nephrogenesis from the kidney development literature provided in Supplementary Table 4. Kidney disease genes are provided in Supplementary Data 9.

### Joint analyses of organoids from multiple donors and between stages

Clusters, particularly representing interstitial cell types, segregated by donor origin when default analyses as described was performed. To identify similar cell types among organoids from multiple donors, we coembedded the cells using canonical correlation analyses using Seurat’s “MultiCCA” function. Twenty canonical components were used for clustering. Clusters were assigned identity based on expression of literature-curated marker genes as well as verified by differential expression analysis for individual lines (AS or ThF). We also used the “MultiCCA” function to co-embed organoids at days 29, 32 and 51, and cluster at a resolution of 1. A similar approach was also used to combine ThF organoids from days 0, 7 and 15.

### Analysis of mouse transplants

For cells from the mouse transplants, we aligned the sequenced reads to a reference combining the human and mouse transcriptomes using the Cellranger software as described before. Multimapped reads were discarded and two expression matrices, representing mouse and human barcodes were derived. In case of barcodes that remained in both matrices after quality filtering, we assigned their identities based on the transcriptome that yielded the higher number of total UMIs. Downstream analysis was performed as described above.

### Analysis of human fetal data

Trimester 1 human fetal kidney single-cell transcriptomes were downloaded from the Data Supplement in Young et al.^27^. Trimester 2 Human fetal kidney single-cell transcriptomes were downloaded from Lindström et al.^28^ (NCBI GEO GSE112570). In both cases, the data were available in the format of gene expression count matrices, which were directly plugged into cell-type identification and assignment routines as described above.

### Assessment of cell-type proportions and compositional differences

Cell-type proportions for each sample were computed as the proportions of cells representing a cell type divided by the total number of cells analyzed for that sample. The Jenson–Shannon Divergence was computed between any two vectors of cell-proportions using the “JSD” function in the *philentropy* R package^71^, using the default log2 transformation. The AS, passage 1 (D7, D15, D29) was excluded when computing summary statistics as it was an outlier in terms of extremely low nephron numbers. ThF, passage 1 (D7) was also excluded as it did not undergo nephrogenesis.

### Comparison of cell types between organoids and human kidney cells

We compared organoid cell with human kidney cells using three approaches:

(1) We used the R package randomForest^72^ to train a multiclass Random Forest classifier (5000 trees) on the mature ThF organoids. The number of training cells per cell type was set at 1000 or 70% of cluster membership, whichever was lower. The remaining cells comprised the test set. The classifier was then used to predict cell types in fetal kidney cells derived from individual kidneys at trimesters one and two, and adult human kidney cells. We inferred cell types for fetal and adult cells using unsupervised clustering as described earlier. Prediction outcomes and concordance of cell types were visualized by a dotplot representation, where *x*-axis levels were ThF cell types and *y*-axis levels were input (fetal or adult) cell types. Each dot (size and color) represented the percentage of cells in the input (*y*-axis level) cell types predicted to be a ThF (*x*-axis level) cell types. The supervised classification analyses revealed high concordance between organoid and human kidney nephron epithelial and podocyte cell types.

(2) We computed the Spearman correlation coefficient between average cluster log2 transformed gene expression profiles in organoid and human samples on a subset of genes defined by the union of the highly variable genes derived independently for organoid, fetal, and human adult.

(3) We coembedded mature organoid profiles from day 29 with fetal or adult data using the “MultiCCA” function in Seurat as described earlier, and derived proportions to infer co-clustering.

### Plotting and visualization

The R package *ggplot2*^73^ was used for visualization of tSNEs, cell-type proportions, boxplots and dotplots. In the dotplot representation, each dot size represented the percentage of cells and the color represented the average nonzero gene expression in log2 scale. We use the *corrplot*^74^ R package for computation and visualization of correlation plots. Wherever heatmaps were used, the average expression of genes across all cells in a cluster (and between replicated) were first computed and then row-scaled to derive *z*-scores and visualized using the *pheatmap*^75^ R package.

### Statistics and reproducibility

All representative images reflect a minimum of three biological replicates.

### Reporting summary

Further information on research design is available in the Nature Research Reporting Summary linked to this article.

## Supplementary information

Supplementary Information

Peer Review File

Description of Additional Supplementary Files

Supplementary Data 1

Supplementary Data 2

Supplementary Data 3

Supplementary Data 4

Supplementary Data 5

Supplementary Data 6

Supplementary Data 7

Supplementary Data 8

Supplementary Data 9

Supplementary Data 10

Supplementary Data 11

Reporting Summary

## Data Availability

The authors declare that all data supporting the findings of this study are available within the article and its supplementary information files or from the corresponding author upon reasonable request. The sequencing data that support the findings of this study are deposited in the GEO database under accession code GSE136314 (AS, ThF), and the controlled-access data repository, Broad DUOS (N1, N2). Processed data are available at the Human Cell Atlas portal (https://singlecell.broadinstitute.org/single_cell/study/SCP211/human-kidney-organoids-atlas). The germ-layer signatures were obtained from Tsankov et al. (ref. ^38^). Public Dataset (NCBI GEO ID: GSE112570) was used for Trimester 2 human fetal analysis. Trimester 1 human fetal kidney single-cell transcriptomes were downloaded from the Data Supplement in Young et al. (ref. ^27^. For raw data see: EGAS00001002171, EGAS00001002486, EGAS00001002325 and EGAS00001002553).

## Code availability

Associated code is available at: https://github.com/ayshwaryas/2019-kidneyorganoid-scrnaseq-reproducibility
