# Supplementary material for: Single cell census of human kidney organoids shows reproducibility and diminished off-target cells after transplantation
Source: Nat Commun. 2019 Nov 29;10:5462. doi: 10.1038/s41467-019-13382-0 (PMC6884507; doi:10.1038/s41467-019-13382-0)
Supplement: Supplementary file 1 — Supplementary Information [file 41467_2019_13382_MOESM1_ESM.pdf]

Supplementary Figure 1

A

Line	Age	Gender	Tissue Origin	iPS vectors
**AS**	NA	Male	Bone marrow CD34+ mononuclear cells	Episomal
**N1**	12	Female	Erythroblasts	Sendai virus
**N2**	46	Male	Erythroblasts	Sendai virus
**ThF**	NA	Female	CD34+ cord blood	Episomal

B

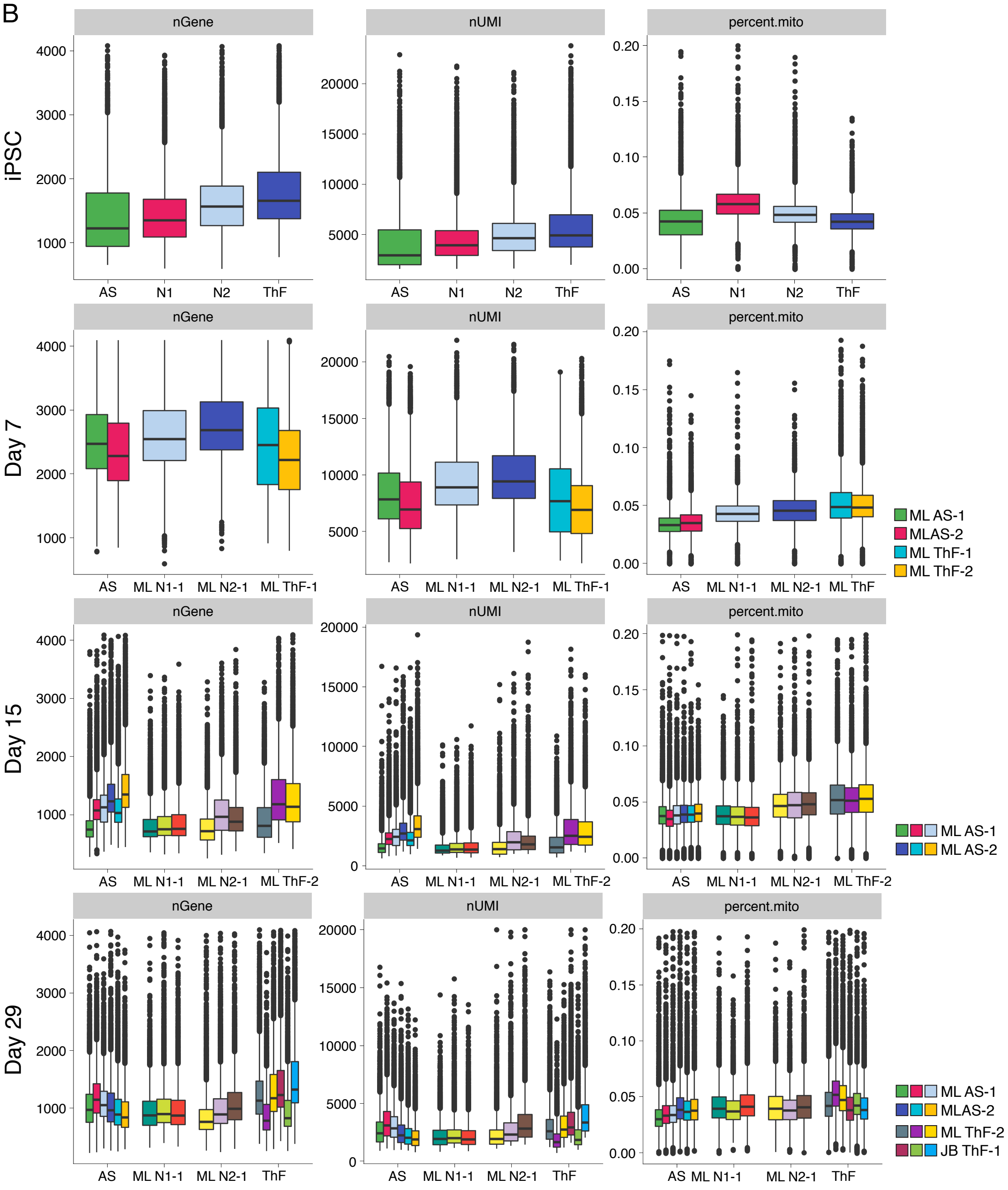

Supplementary Figure 1. Data quality metrics for single cell analysis of kidney organoids across four iPSC lines. (A) Table of reference for 4 different iPSC lines. (B) Boxplots of quality control metrics (nGene: number of genes with a normalized expression value above 0 per cell, lower QC cutoff 200; nUMI: the total number of Unique Molecule Identifiers (UMIs) detected per cell, lower QC cutoff 1000; percent.mito: the proportion of reads mapping to mitochondrial genes, upper QC cutoff of 20%) across cell lines and time points. The center line of the boxplots indicates the median, the bottom and top lines of the box indicate the first and third quartiles respectively of the data points. Outliers are indicated as dots beyond the whiskers; whiskers stretch up to +-1.5*IQR on both sides. Each data point is a QC measure for a cell.

Supplementary Figure 2

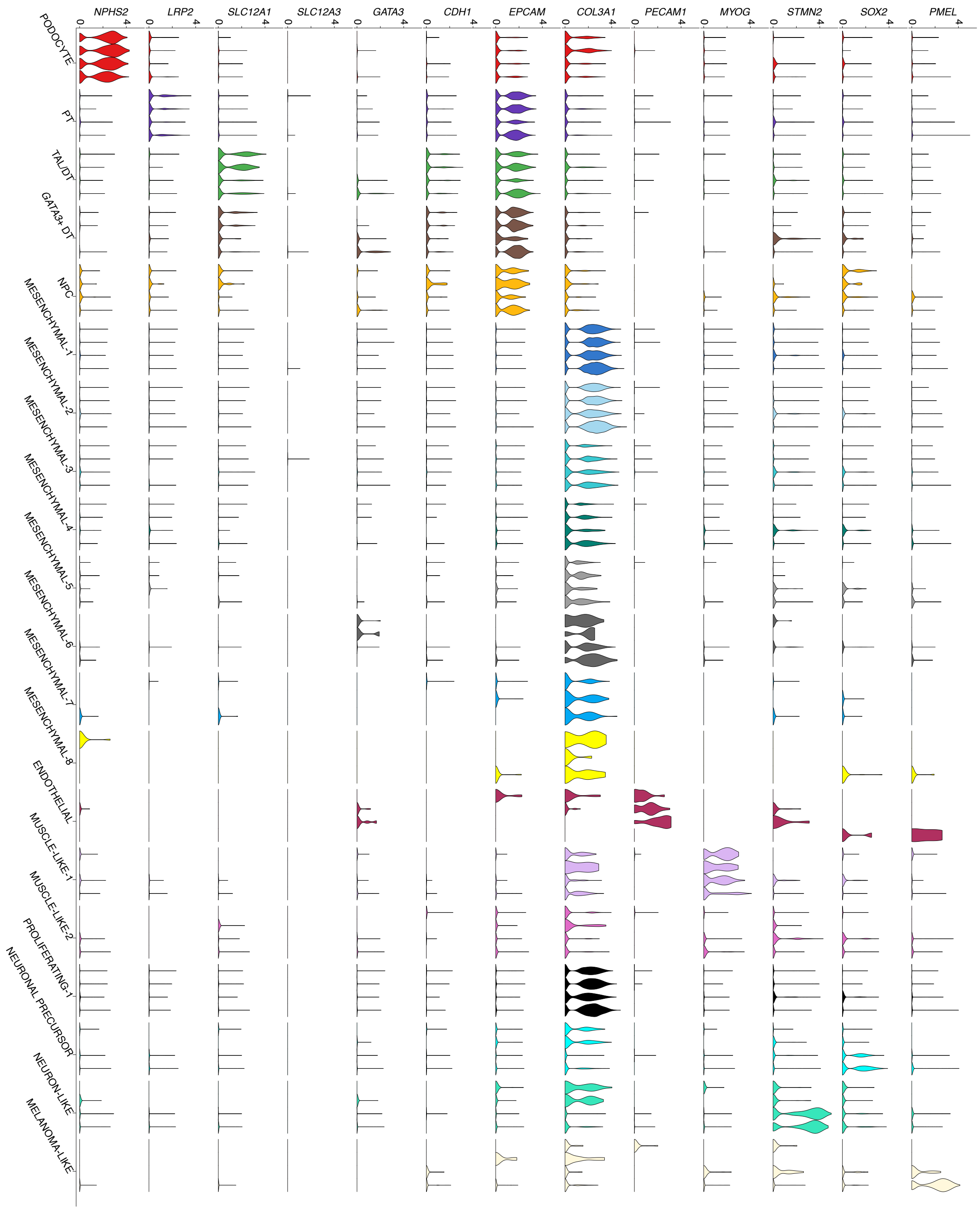

Supplementary Figure 2. D29 kidney organoids express canonical markers of major nephron, mesenchymal and off-target cell types.

Violin plots of single cell gene expression from D29 kidney organoids for canonical markers. X-axis annotations represent cell types. Each violin per cell type represents an iPSC line of origin, AS, N1, N2 and ThF in order from left to right. PT, Proximal Tubule; TAL, Thick Ascending Limb; DT, Distal Tubule; NPC, Nephron Progenitor Cell.

Supplementary Figure 3

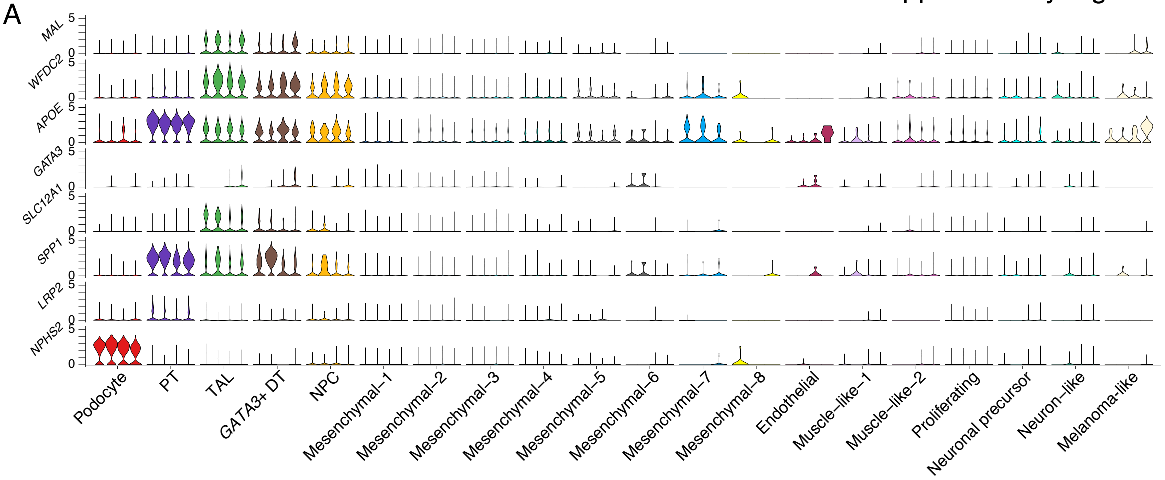

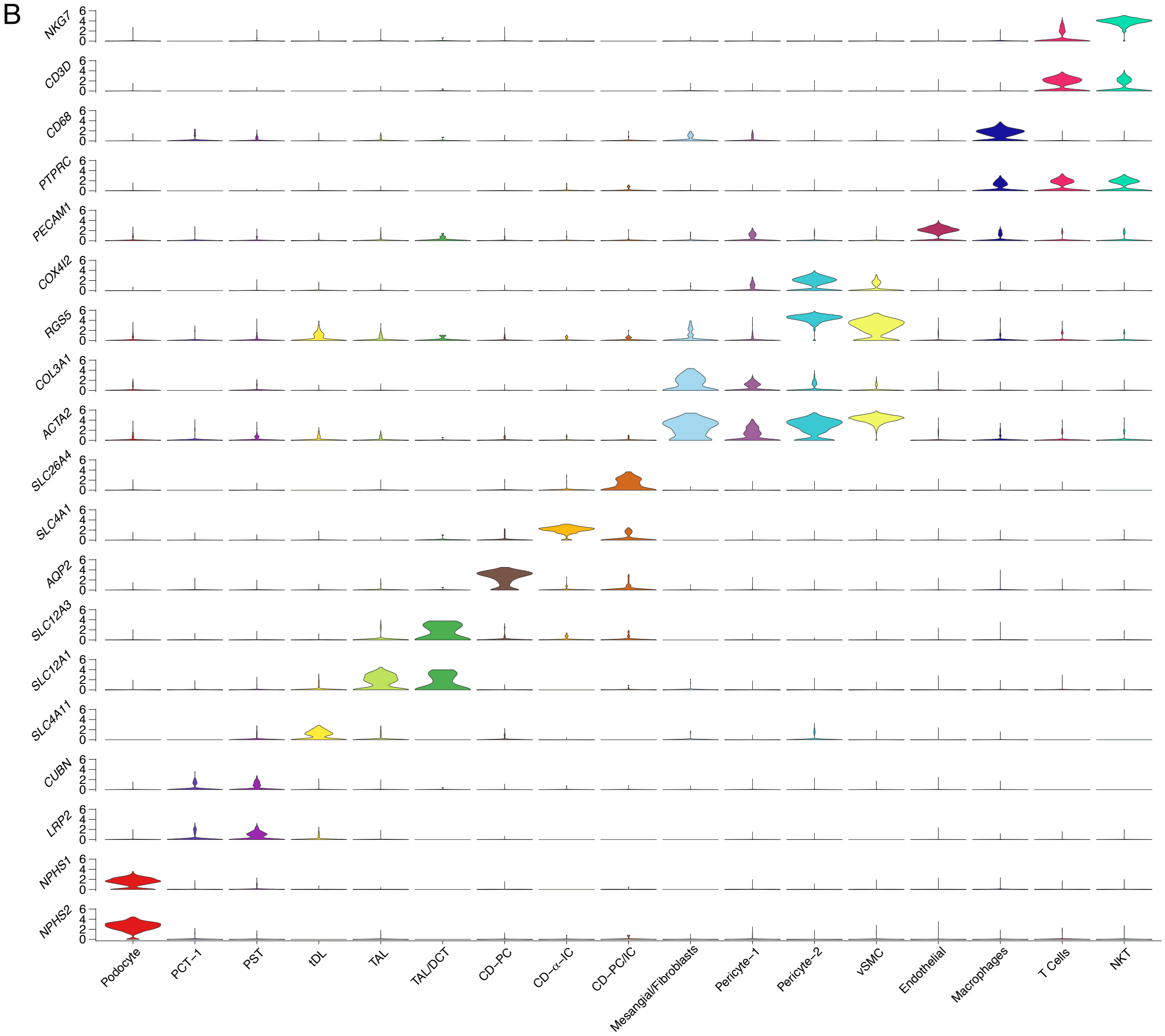

Supplementary Figure 3. D29 data-derived markers and canonical markers of major human nephron and kidney cell types.

(A) Violin plots of single cell gene expression from D29 kidney organoids for data-driven markers. X-axis annotations represent cell types. Each violin per cluster represents a line, AS, N1, N2, and ThF in order from left to right. (B) Violin plot of single cell gene expression from human adult nephrectomy for canonical markers. X-axis annotations represent cell types. PCT, Proximal Convoluted Tubule; PST, Proximal Straight Tubule; tDL, thin descending limb; DCT, distal convoluted tubule; CD, Collecting Duct; PC, Principal Cell; IC, Intercalated Cell; vSMC, vascular smooth muscle cell; NKT, Natural Killer T cell.

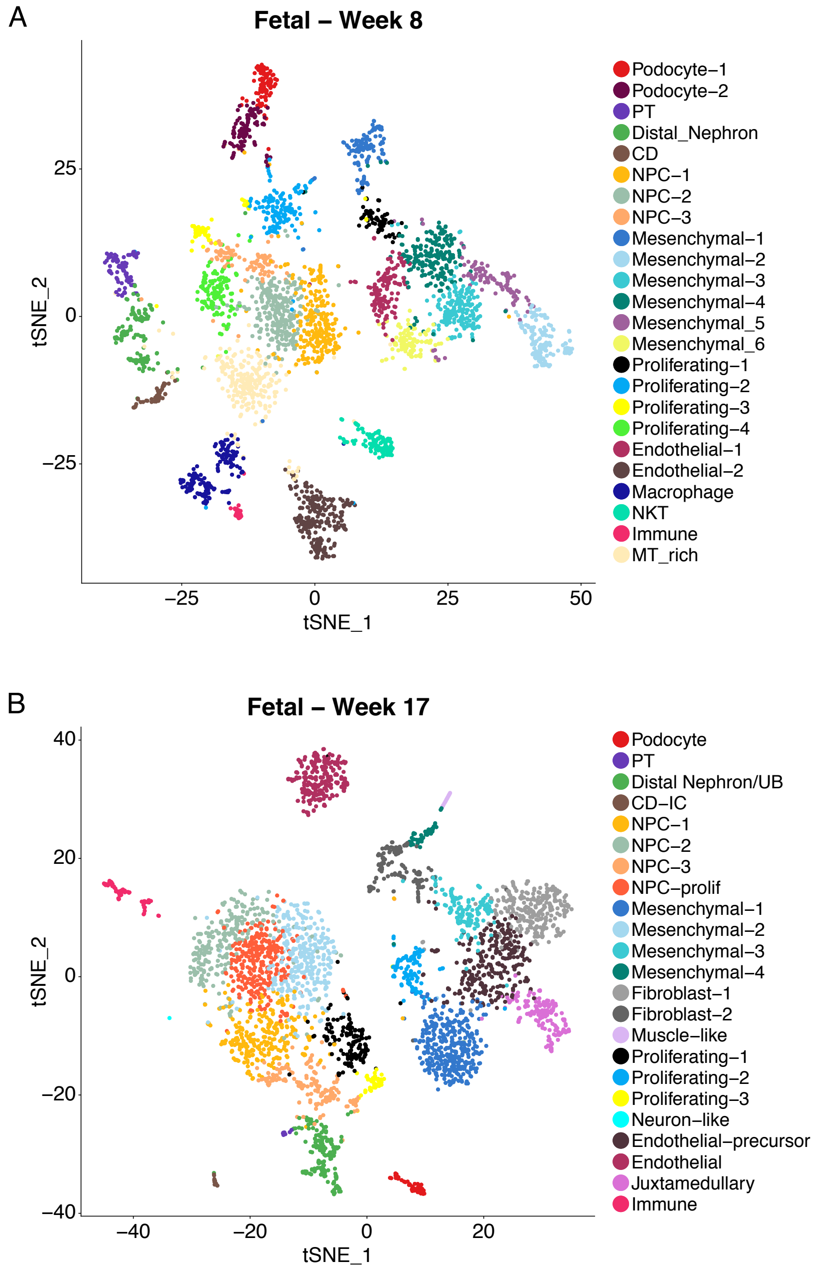

Supplementary Figure 4. First and second trimester fetal kidney.
t-SNE plot of single cell transcriptomic profiles from (A) first (Week 8) and (B) second (Week 17) trimester human fetal kidney. Cluster color annotations as shown. MT, mitochondrial. UB, ureteric bud.

Supplementary Figure 5

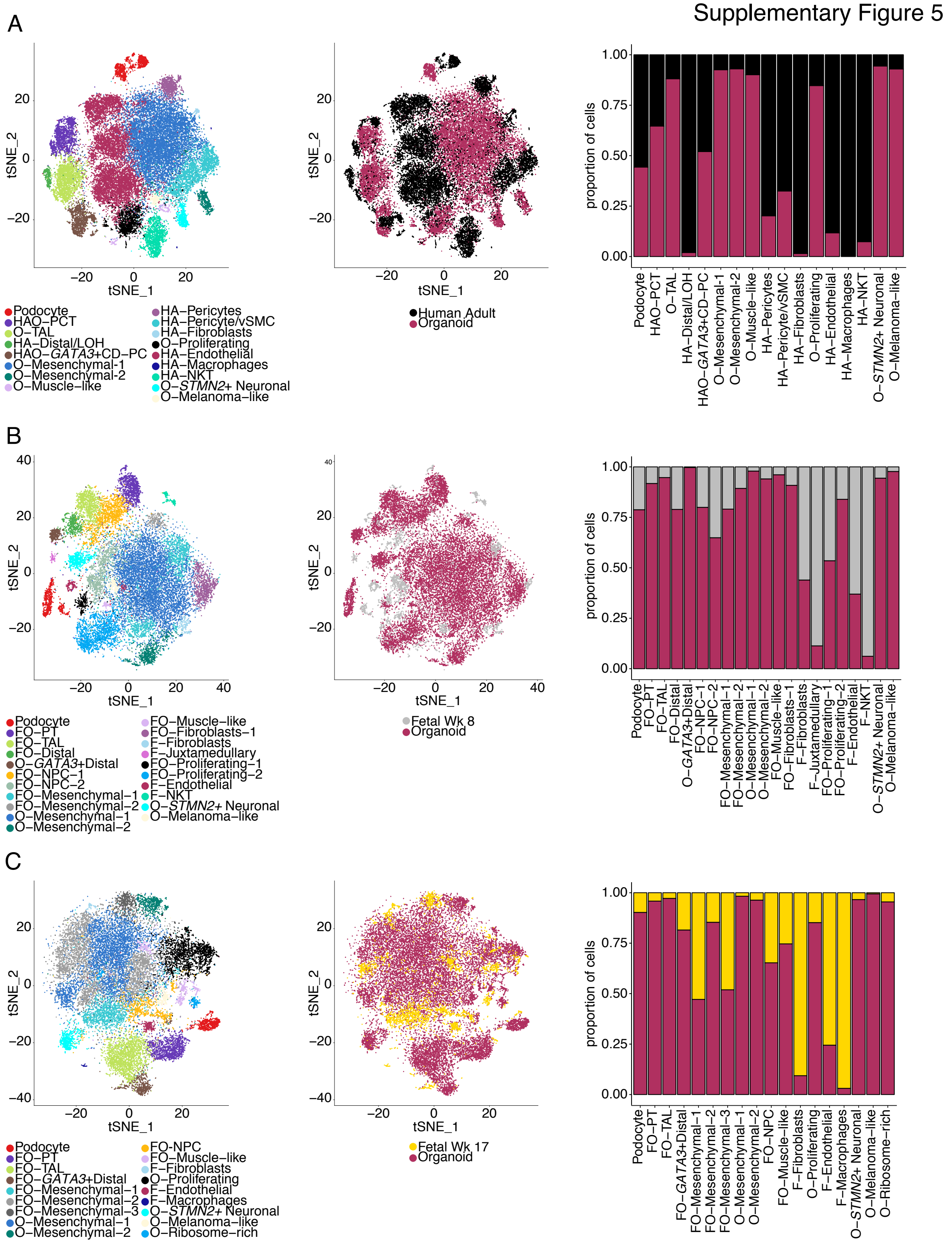

Supplementary Figure 5. Integrated analysis of human adult and fetal data with D29 organoids.

Integrated analysis of D29 organoid with (A) human adult and (B) first (Wk 8) and (C) second (Wk 17) trimester fetal kidney data. (Left) t-SNE plot of single cell transcriptomic profiles with cell type color annotations; (Middle) t-SNE plot of single cell transcriptomic profiles with sample origin color annotations; (Right) Proportion plots of sample contribution per cell type. HA, Human Adult; O, Organoid; HAO, well-represented in both human adult and organoid; F, Fetal; FO, well-represented in both fetal and organoid stages.

Supplementary Figure 6

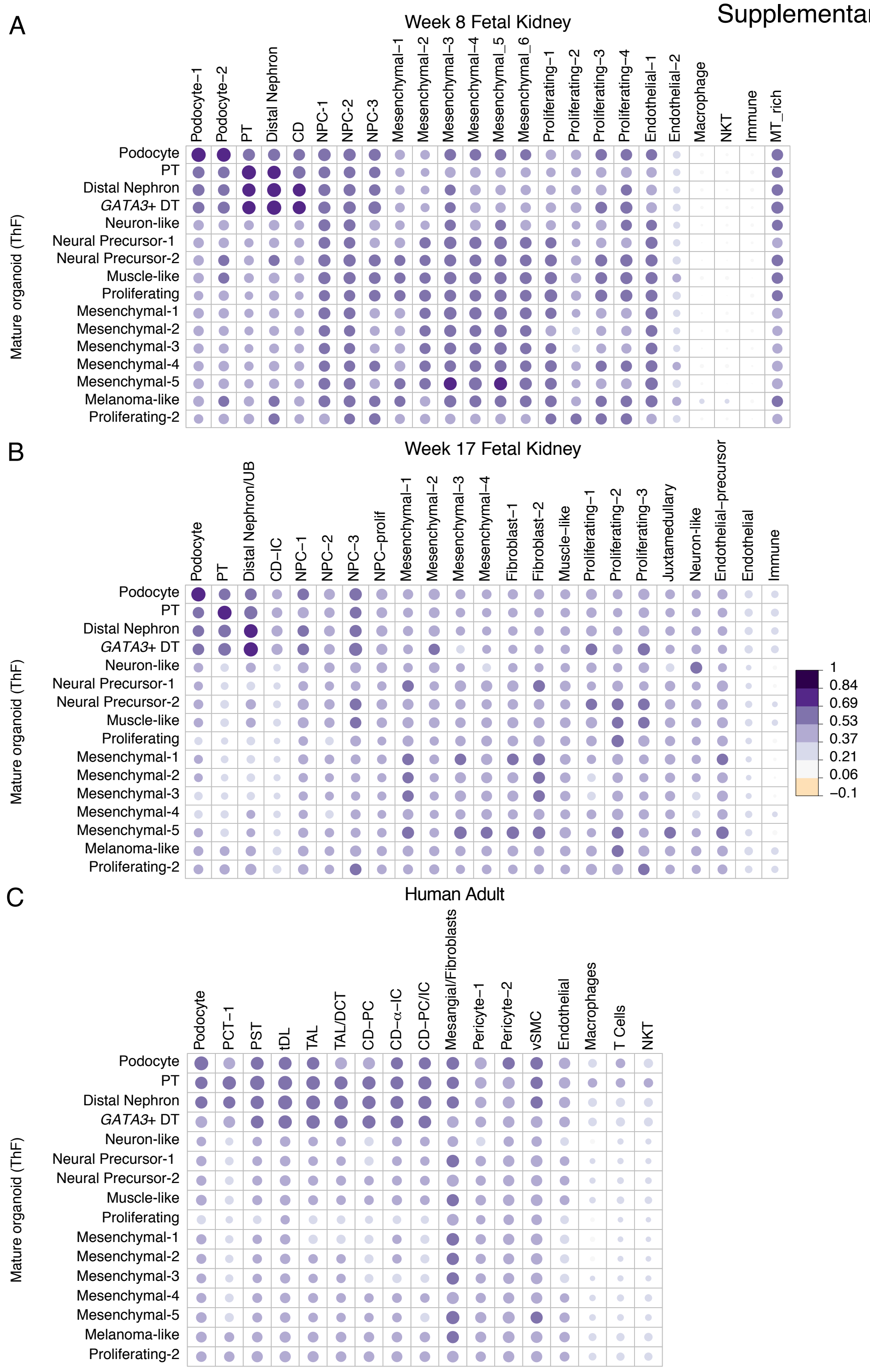

Supplementary Figure 6. Spearman correlation between human adult and fetal data versus D29 organoids.
Spearman correlation coefficient of D29 organoid cell types (ThF line) versus (A) first (Wk 8) and (B) second (Wk 17) trimester fetal kidney and (C) human adult clusters.

Supplementary Figure 7

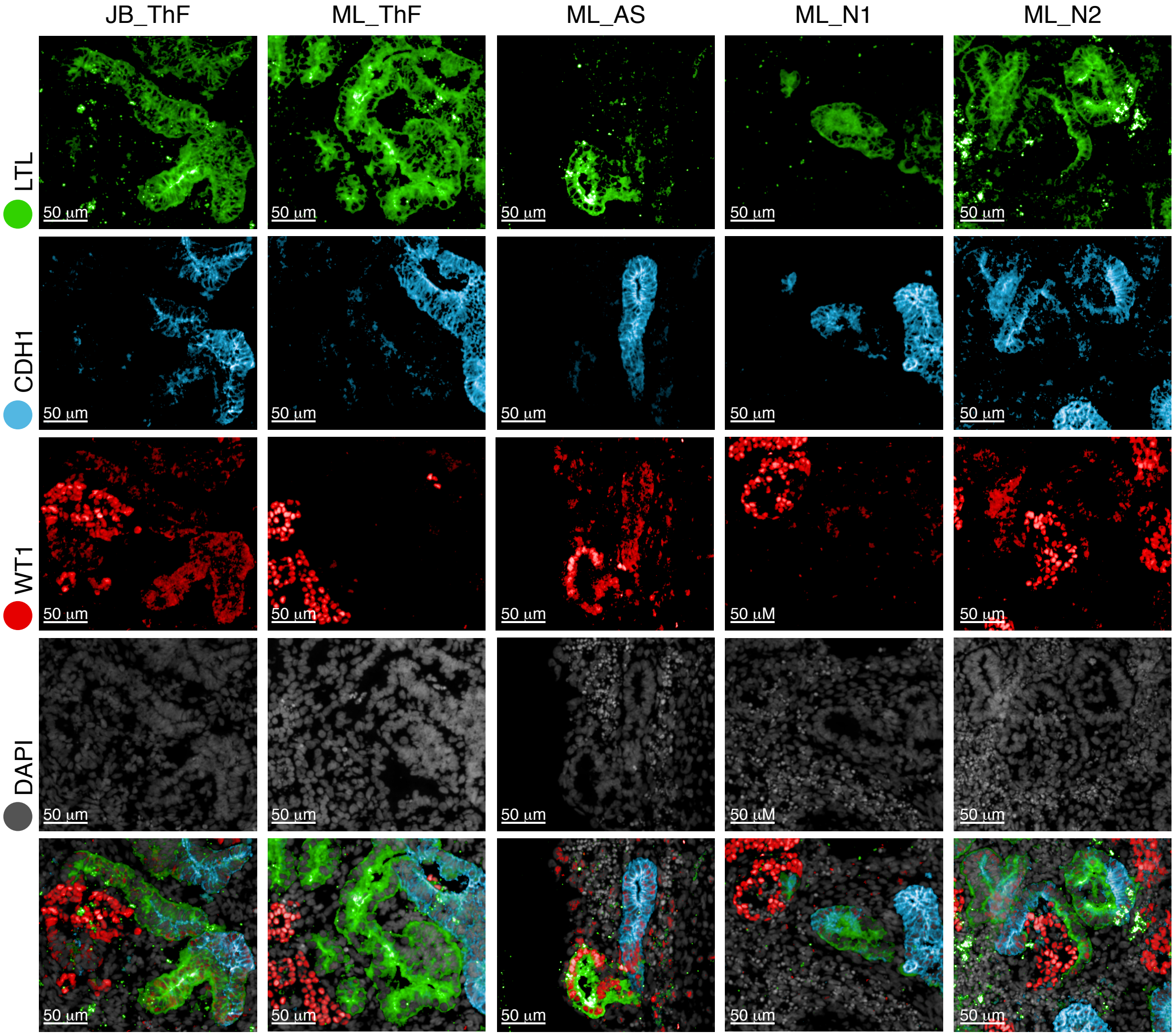

Supplementary Figure 7. Immunofluorescence analysis of D29 kidney organoids demonstrates presence of major nephron cell types from glomerulus to distal tubule. Immunofluorescence staining of D29 kidney organoids for podocytes (WT1), proximal tubular cells (LTL), and the distal tubular compartment (ECAD) across two protocols (JB, ML) and four cell lines (ThF, AS, N1, N2).

Supplementary Figure 8

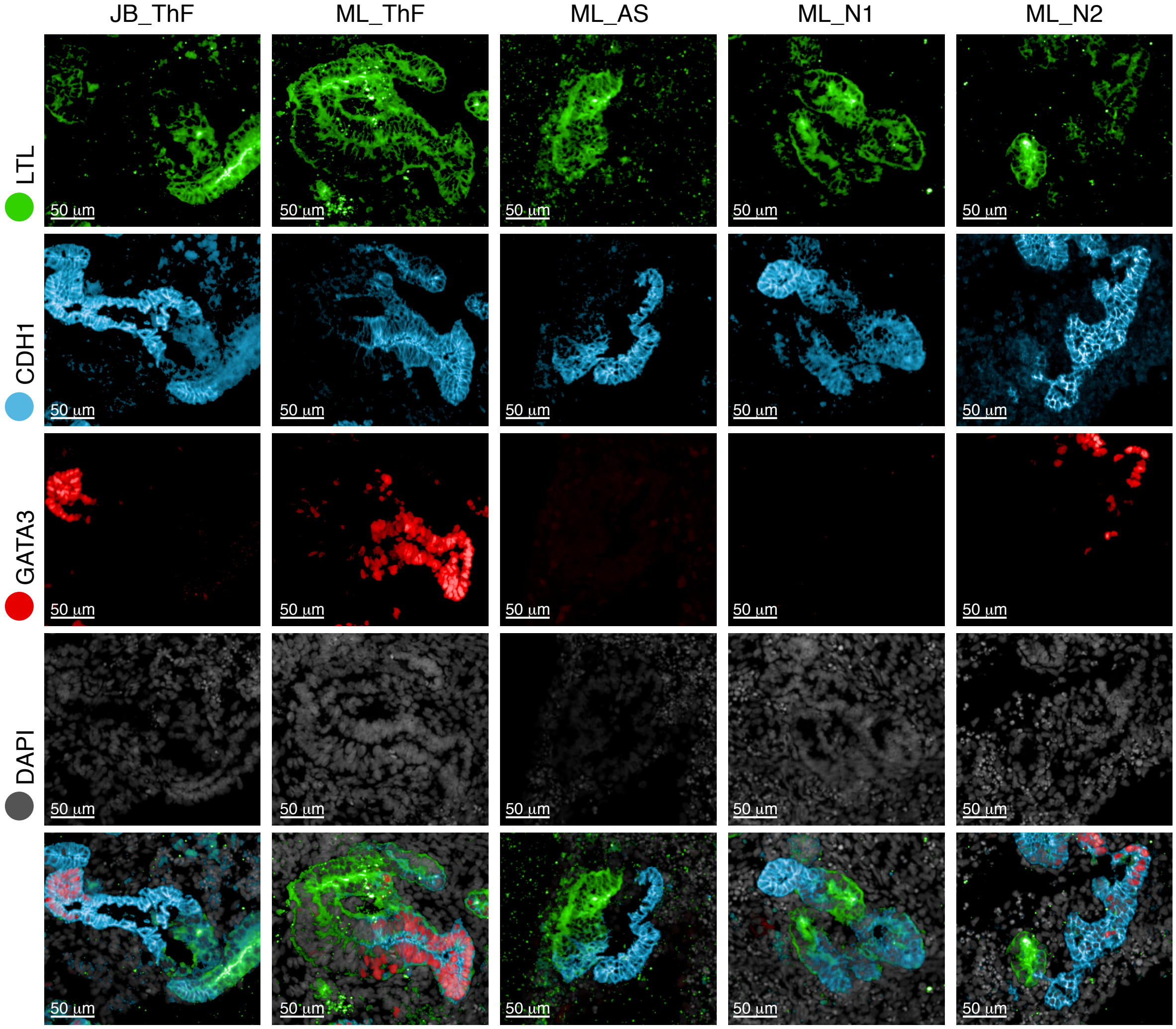

Supplementary Figure 8. Immunofluorescence analysis of D29 kidney organoids demonstrates presence of major kidney epithelial cell types from proximal to distal tubule. Immunofluorescence staining of D29 kidney organoids for proximal tubule (LTL), and distal nephron compartment (ECAD, GATA3) across two protocols (JB, ML) and four cell lines (ThF, AS, N1, N2).

Supplementary Figure 9

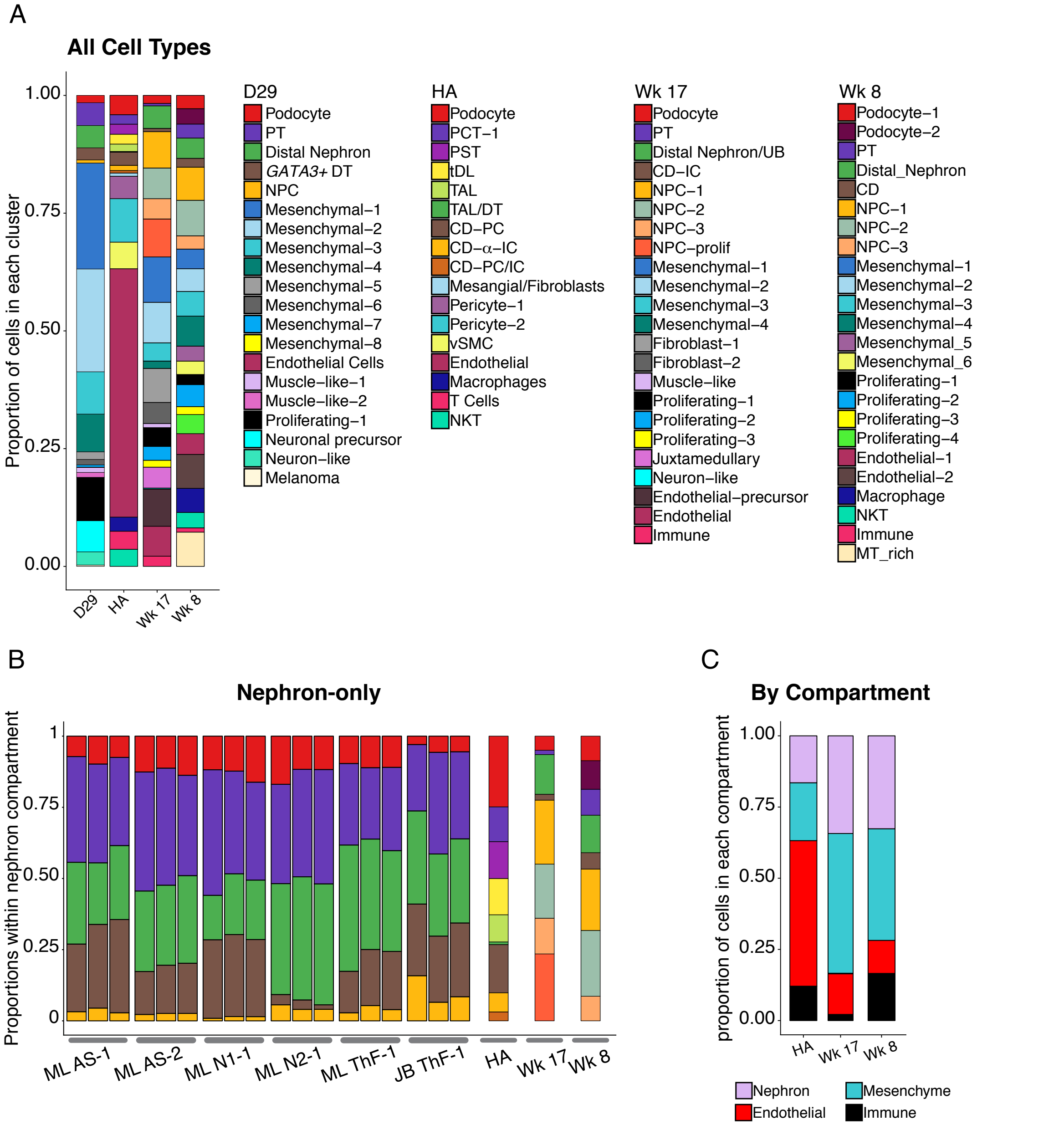

Supplementary Figure 9. Cell type proportions of D29 kidney organoids in comparison to human adult and fetal kidneys.
(A) Proportions of cells per cell type averaged across all D29 organoid samples, human adult (HA), and first (Wk 8) and second (Wk 17) trimester fetal kidneys. (B) Proportions of cell classes within the nephron compartment across all lines and replicates at D29, and human adult (HA), and first (Wk 8) and second (Wk 17) trimester fetal kidneys. (C) Proportions of cell compartments across human adult (HA), and first (Wk 8) and second (Wk 17) trimester fetal kidneys.

A

Day 0 - Cell cluster signature

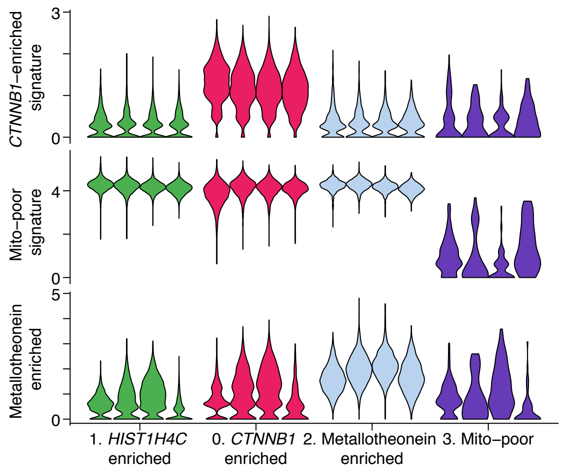

Cluster	Genes	
CTNNB1-enriched	*CTNNB1, SOX11, CDC42, PTPRS*	↑
Metallotheonein-enriched	*MT1H,MT1G,MT1X*	↑
Mito-poor	*MT-CO3, MT-CYB, MT-ND4*	↓

B

Day 7 - Cell cluster signature

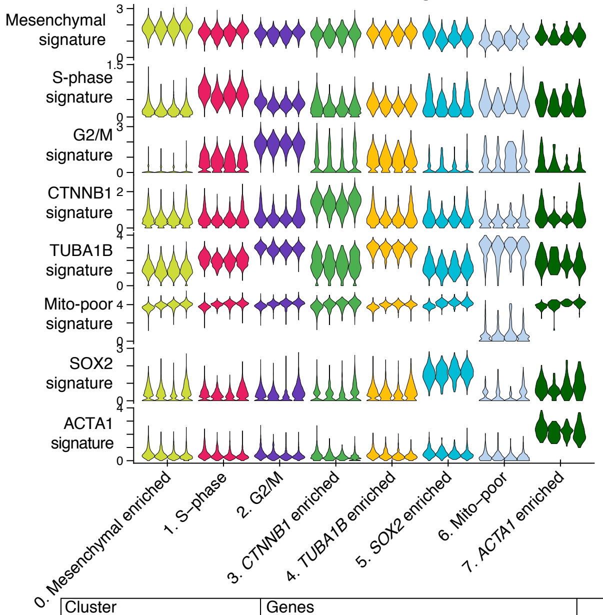

Cluster	Genes	
Mesenchymal enriched	*ACTG1, DLK1, BST2,WT1*	↑
S-phase enriched	*RPA2,MCM4,RFC4,MCM6,PCNA,MCM5, LIG1,RNASEH2A,MCM7,RFC2,RPA3, MCM3,FEN1*	↑
G2/M enriched	*TOP2A, CENPF*	↑
CTNNB1 enriched	*CTNNB1, SOX11, CDC42, PTPRS*	↑
TUBA1B enriched	*TUBA1B,PTTG1*	↑
SOX2 enriched	*SOX2,HOXD1,TGFBI*	↑
Mito-poor	*MT-CO3, MT-CYB, MT-ND4*	↓
ACTA1 enriched	*ACTA2,MYL9,TAGLN,CTGF,ACTC1, TPM1,ANXA2, SPARC*	↑

Supplementary Figure 10. Cluster specific gene signatures for iPSC D0 and D7 stages
(A) Violin plot of average expression of cluster specific gene signatures in cells in each line at the iPSC stage. Signatures are provided in the table. (B) Violin plot of average expression of cluster specific gene signatures in cells in each line at D7. Signatures are provided in the accompanying table. In each cluster, violins for AS, N1, N2 and ThF are in order from left to right.

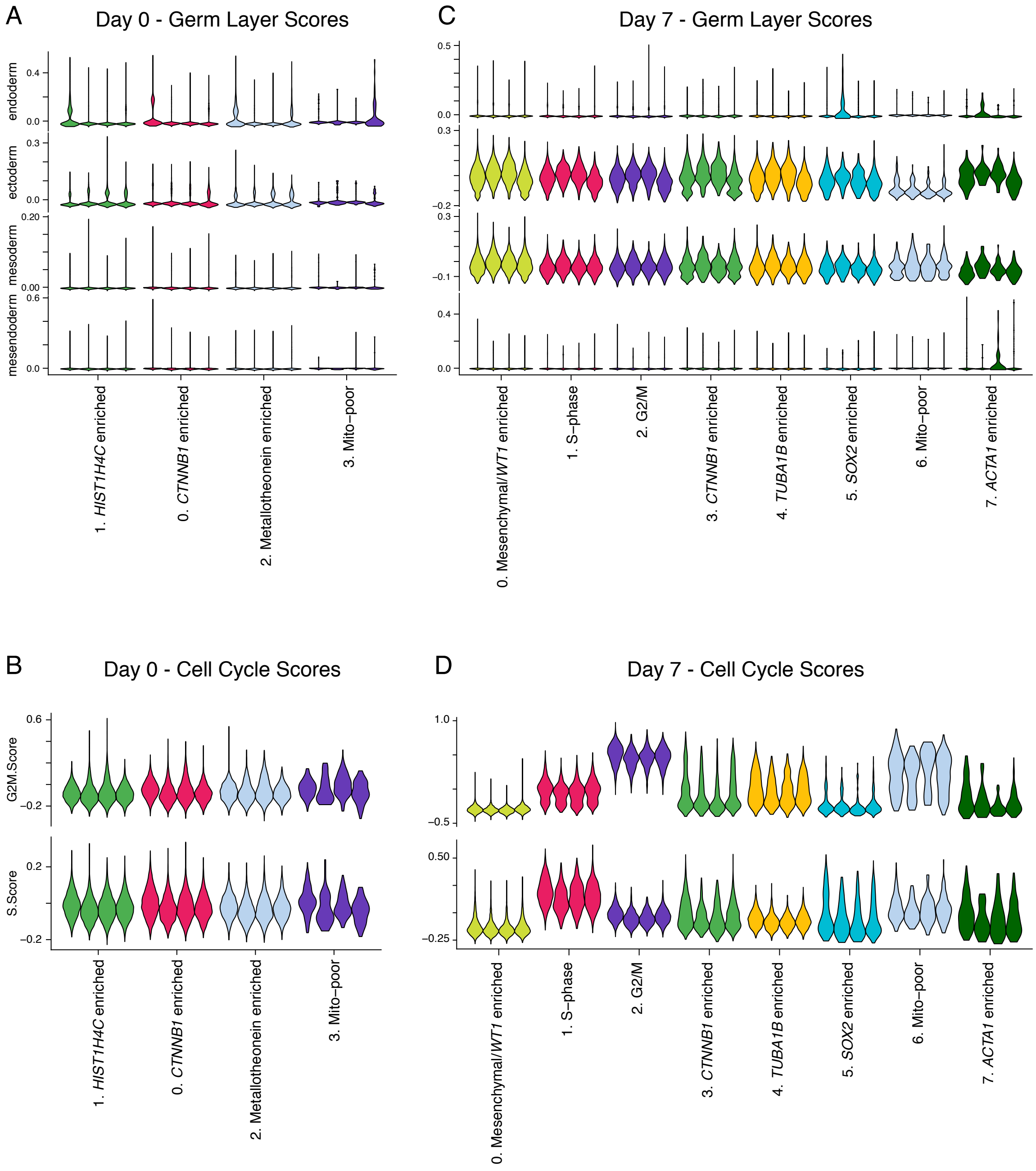

Supplementary Figure 11. Single cell analysis of 4 iPSC lines reveals no priming for any specific germ layer and expression of cell-cycle markers.
Violin plot of cluster-specific average expression of germ-layer signatures in single cells from the 4 iPSC lines at (A) D0 and (C) D7. Violin plot of scores for cell cycle phases G2M and S-phase in cells in each iPSC line at (B) D0 and (D) D7 in a cluster specific manner. In each cluster, violins for AS, N1, N2 and ThF are in order from left to right.

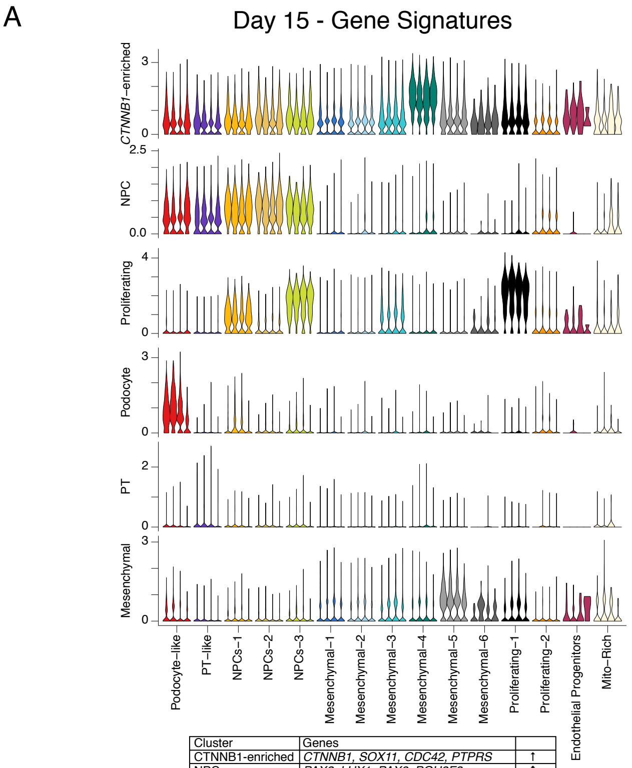

Cluster	Genes	
CTNNB1-enriched	*CTNNB1, SOX11, CDC42, PTPRS*	↑
NPC	*PAX2, LHX1, PAX8, POU3F3*	↑
Proliferating	*TOP2A, CENPF*	↑
Podocyte	*NPHS1, NPHS2, PTPRO, MAFB*	↑
PT	*CUBN, LRP2*	↑
Mesenchymal	*COL3A1, COL1A1, MEIS2*	↑

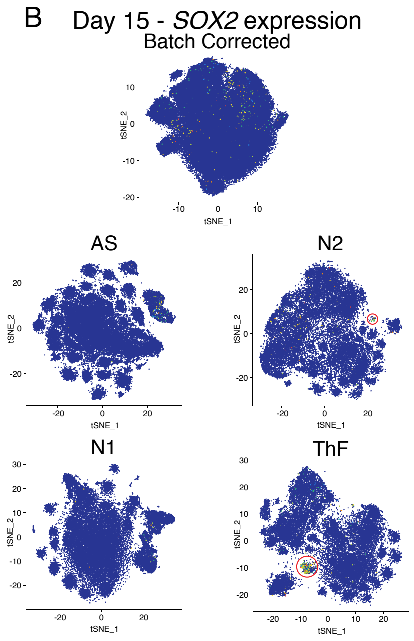

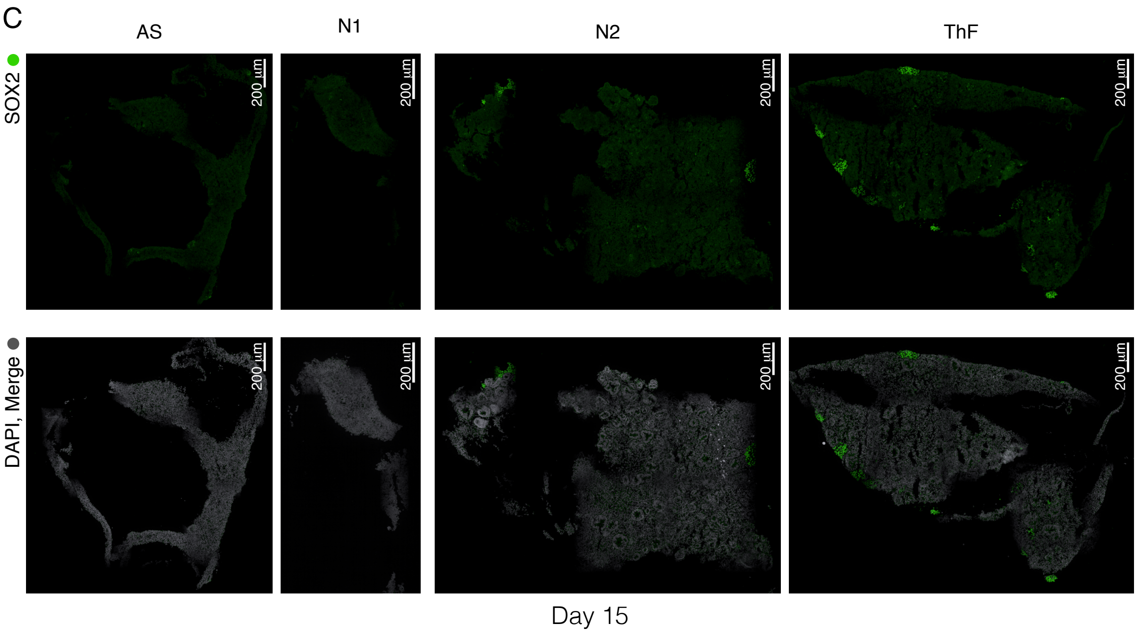

Supplementary Figure 12. Expression of cluster specific gene signatures, line-specific SOX2+ progenitor pools and early markers of podocyte differentiation at D15.
(A) Violin plot of average expression of cluster specific gene signatures in cells in each line at D15 (B) tSNE plot showing variability across the lines in presence of SOX2-positive progenitor cells at D15. Subpopulation of cells (in red circle) expressing SOX2 emerge as early as D15 in ThF and N2. (C) Immunofluorescence staining of D15 kidney organoids for neuron off-target cell populations (SOX2) across four cell lines (AS, N1, N2, ThF).

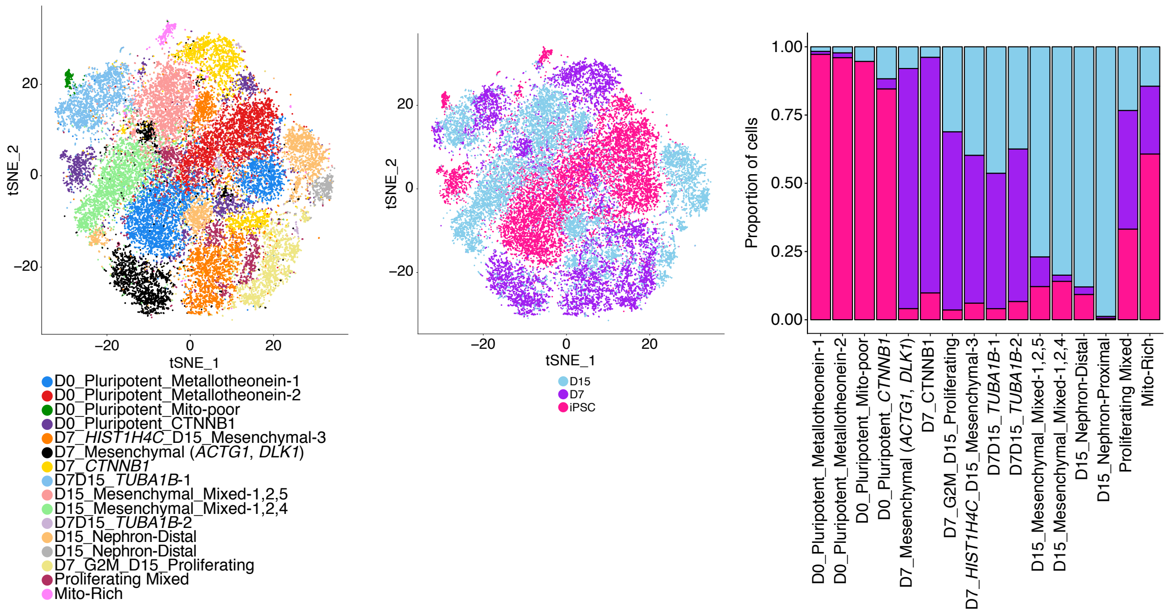

Supplementary Figure 13. Integrated analysis of D0, D7, and D15 kidney organoid data. Integrated analysis of ThF line D0, D7, and D15 kidney organoid data. (Left) t-SNE plot of single cell transcriptomic profiles with cluster color annotations; (Middle) t-SNE plot of single cell transcriptomic profiles with sample origin color annotations; (Right) Proportion plots of sample contribution per cluster.

A

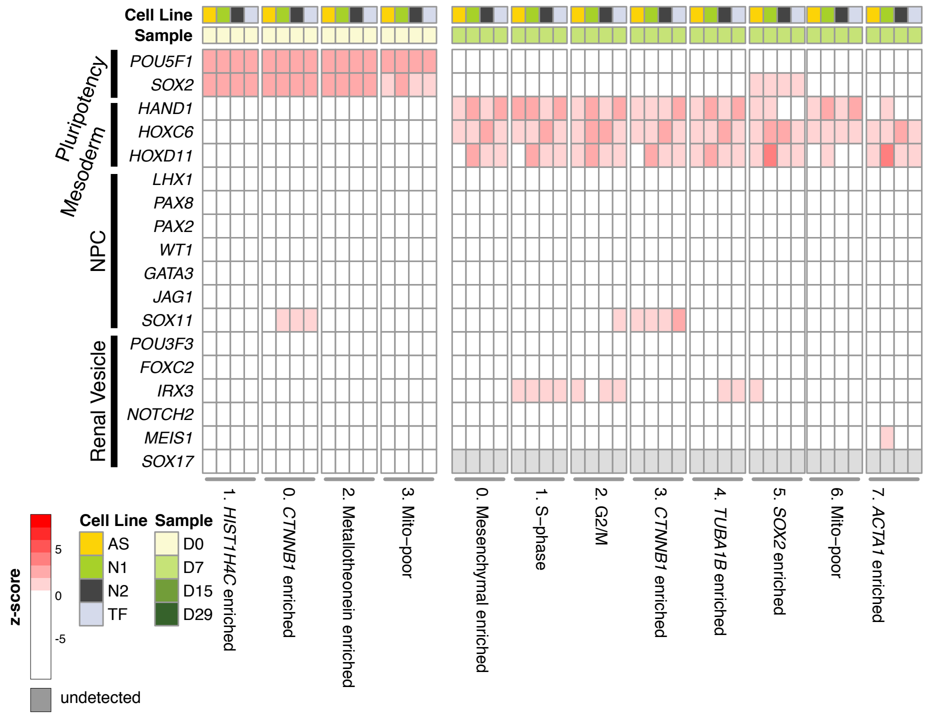
Cell Line
Sample
Pluripotency
Mesoderm
NPC
Renal Vesicle
POU5F1
SOX2
HAND1
HOXC6
HOXD11
LHX1
PAX8
PAX2
WT1
GATA3
JAG1
SOX11
POU3F3
FOXC2
IRX3
NOTCH2
MEIS1
SOX17
1. HIST1H4C enriched
0. CTNNB1 enriched
2. Metallotheonein enriched
3. Mito−poor
0. Mesenchymal enriched
1. S−phase
2. G2/M
3. CTNNB1 enriched
4. TUBA1B enriched
5. SOX2 enriched
6. Mito−poor
7. ACTA1 enriched
z-score
5
0
-5
undetected
Cell Line
AS
N1
N2
TF
Sample
D0
D7
D15
D29

B

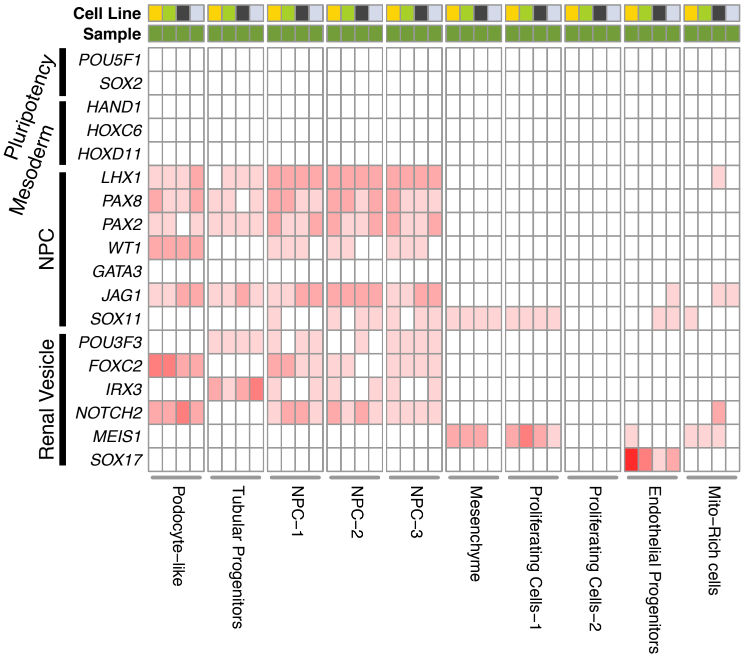
Cell Line
Sample
Pluripotency
Mesoderm
NPC
Renal Vesicle
POU5F1
SOX2
HAND1
HOXC6
HOXD11
LHX1
PAX8
PAX2
WT1
GATA3
JAG1
SOX11
POU3F3
FOXC2
IRX3
NOTCH2
MEIS1
SOX17
Podocyte−like
Tubular Progenitors
NPC−1
NPC−2
NPC−3
Mesenchyme
Proliferating Cells−1
Proliferating Cells−2
Endothelial Progenitors
Mito−Rich cells

Supplementary Figure 14. Kidney organoid differentiation follows kidney nephrogenesis as determined by expression of transcriptional programs across organoid development time.
Heat map comparison of expression of major transcription factors and other canonical markers of nephrogenesis across organoid differentiation, (A) iPSC and D7 (B) D15, across the 4 iPSC lines.

A

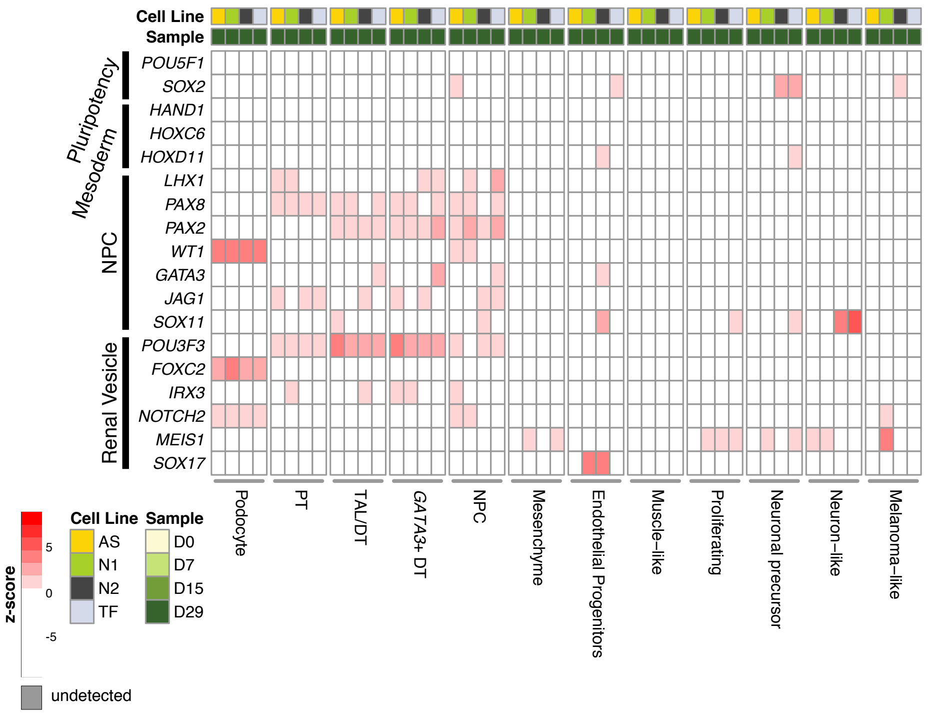
Cell Line
Sample
Pluripotency
Mesoderm
NPC
Renal Vesicle
POU5F1
SOX2
HAND1
HOXC6
HOXD11
LHX1
PAX8
PAX2
WT1
GATA3
JAG1
SOX11
POU3F3
FOXC2
IRX3
NOTCH2
MEIS1
SOX17
Podocyte
PT
TAL/DT
GATA3+ DT
NPC
Mesenchyme
Endothelial Progenitors
Muscle–like
Proliferating
Neuronal precursor
Neuron–like
Melanoma–like
z-score
5
0
-5
Cell Line
AS
N1
N2
TF
Sample
D0
D7
D15
D29
undetected

B

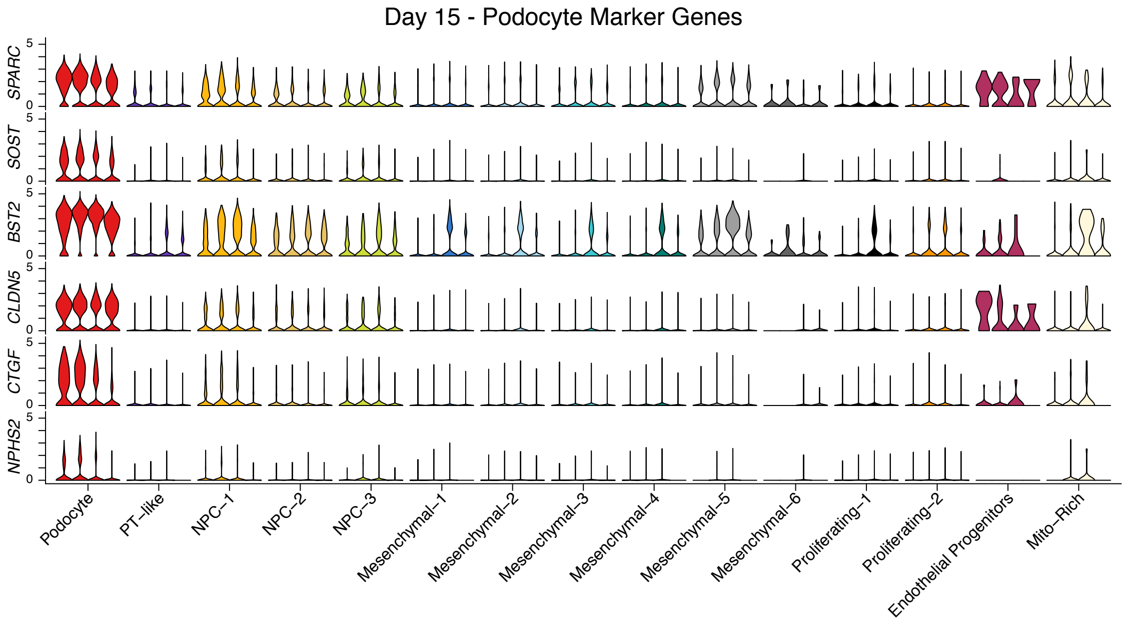
Day 15 - Podocyte Marker Genes
SPARC
SOST
BST2
CLDN5
CTGF
NPHS2
5
0
Podocyte
PT–like
NPC–1
NPC–2
NPC–3
Mesenchymal–1
Mesenchymal–2
Mesenchymal–3
Mesenchymal–4
Mesenchymal–5
Mesenchymal–6
Proliferating–1
Proliferating–2
Endothelial Progenitors
Mito–Rich

Supplementary Figure 15. Kidney organoid differentiation follows kidney nephrogenesis as determined by expression of transcriptional programs across organoid development time and data-driven podocyte marker genes at D15.
(A) Heat map comparison of expression of major transcription factors and other canonical markers of nephrogenesis in D29 organoids across 4 iPSC lines. (B) Violin plots from D15 organoids for expression of canonical (NPHS2) and data-driven (CLDN5, SOST, BST2, SPARC, CTGF) podocyte marker genes. In each cluster, violins for AS, N1, N2 and ThF are in order from left to right.

Supplementary Figure 16

CAKUT

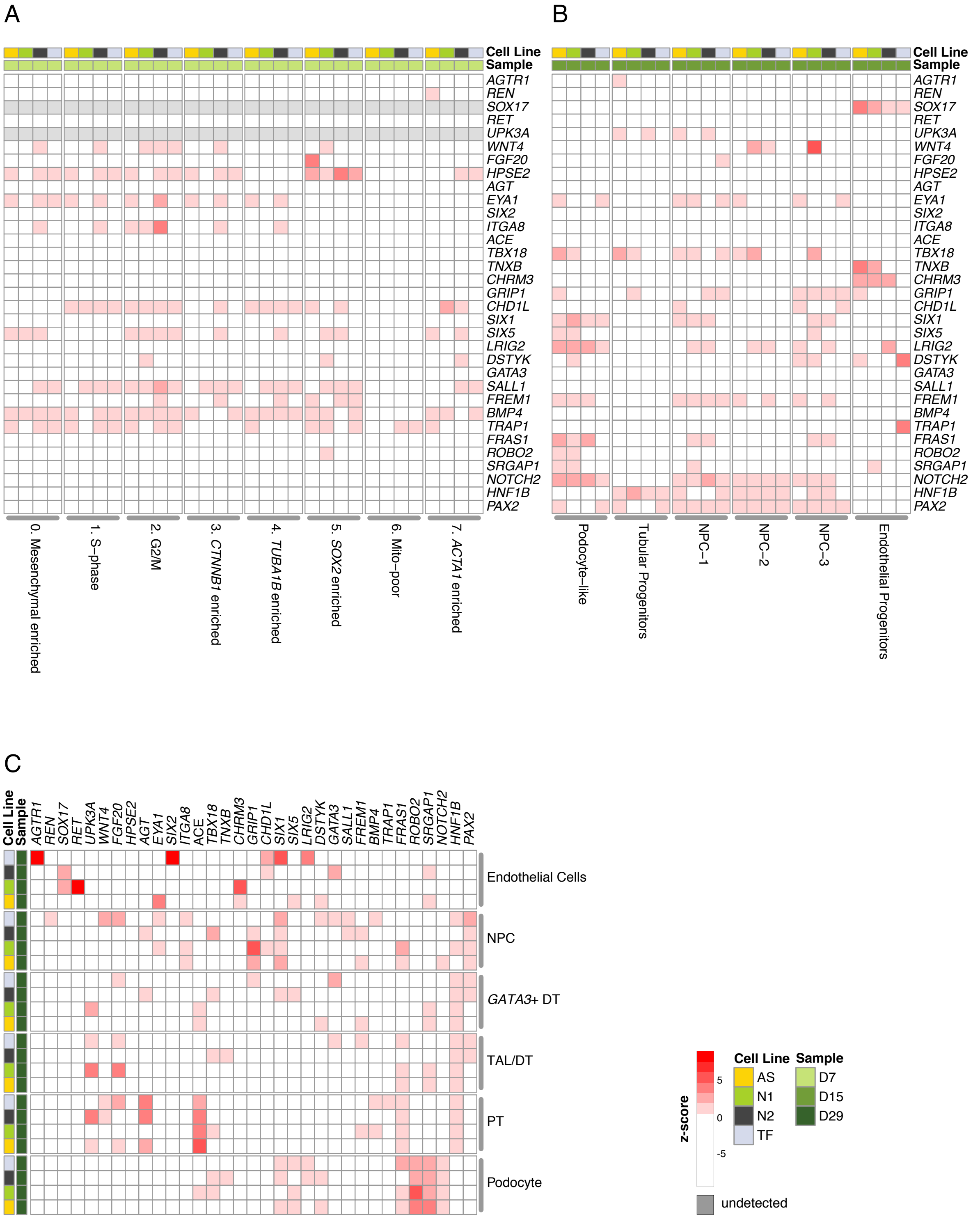

Supplementary Figure 16. Expression of monogenic causes of congenital abnormalities of the kidney and urinary tract (CAKUT) in appropriate nephron epithelial cell types suggests utility of kidney organoids for understanding genetic kidney diseases.
Heat map comparison of gene expression in organoids across differentiation ((A) D7, (B) D15, and (C) D29) of CAKUT-causing genes in kidney epithelial cell clusters (D15, D29) across 4 iPSC lines.

Mendelian Cystic Diseases & Tumor Syndromes

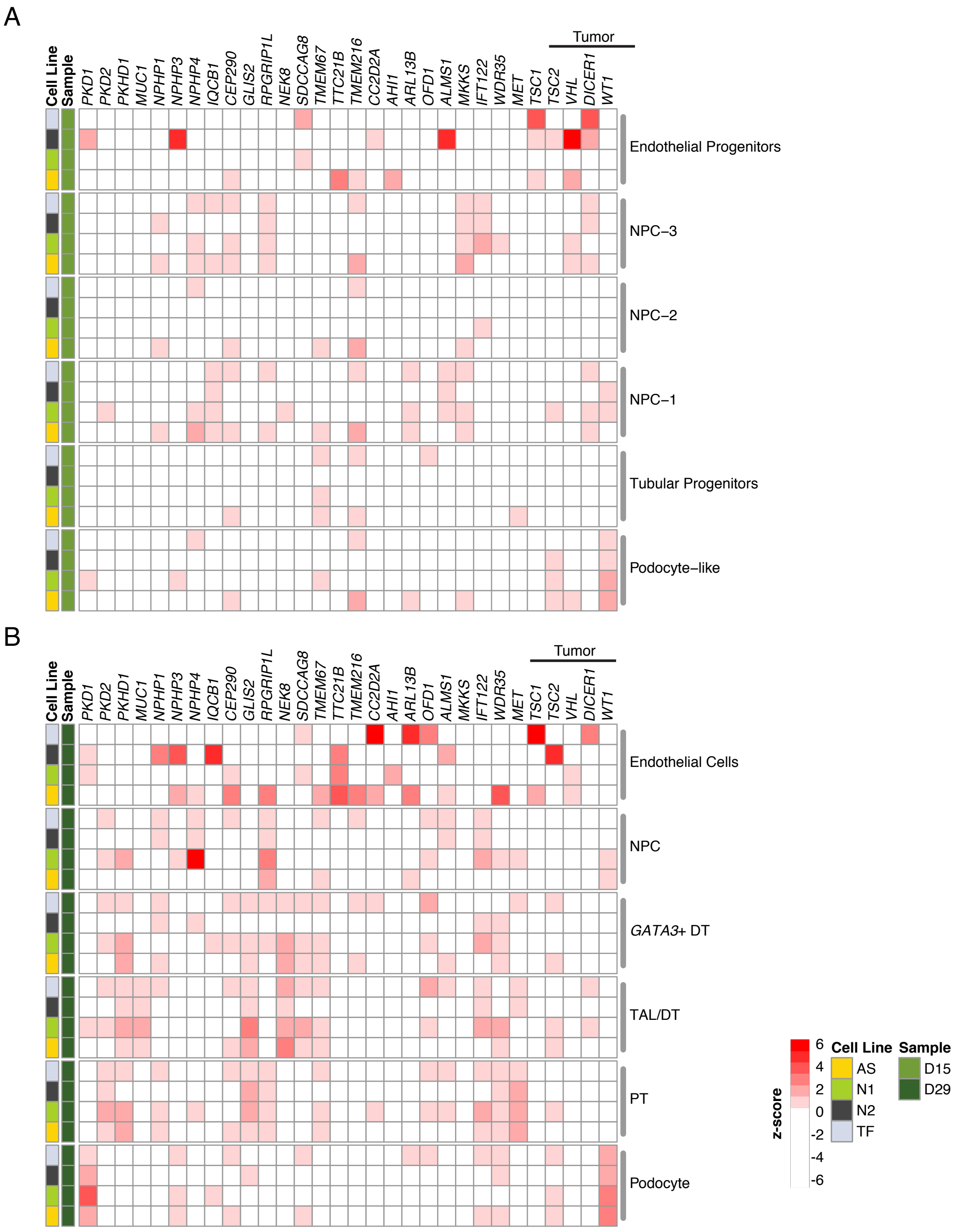

Supplementary Figure 17. Expression of monogenic causes of hereditary renal cystic (HRC) diseases and tumor syndromes in appropriate nephron epithelial cell types suggests utility of kidney organoids for understanding genetic kidney diseases.
Heat map comparison of gene expression in organoids (A) D15, (B) D29 of HRC and tumor syndrome diseases-causing genes in kidney epithelial cell clusters across 4 iPSC lines.

CKD GWAS

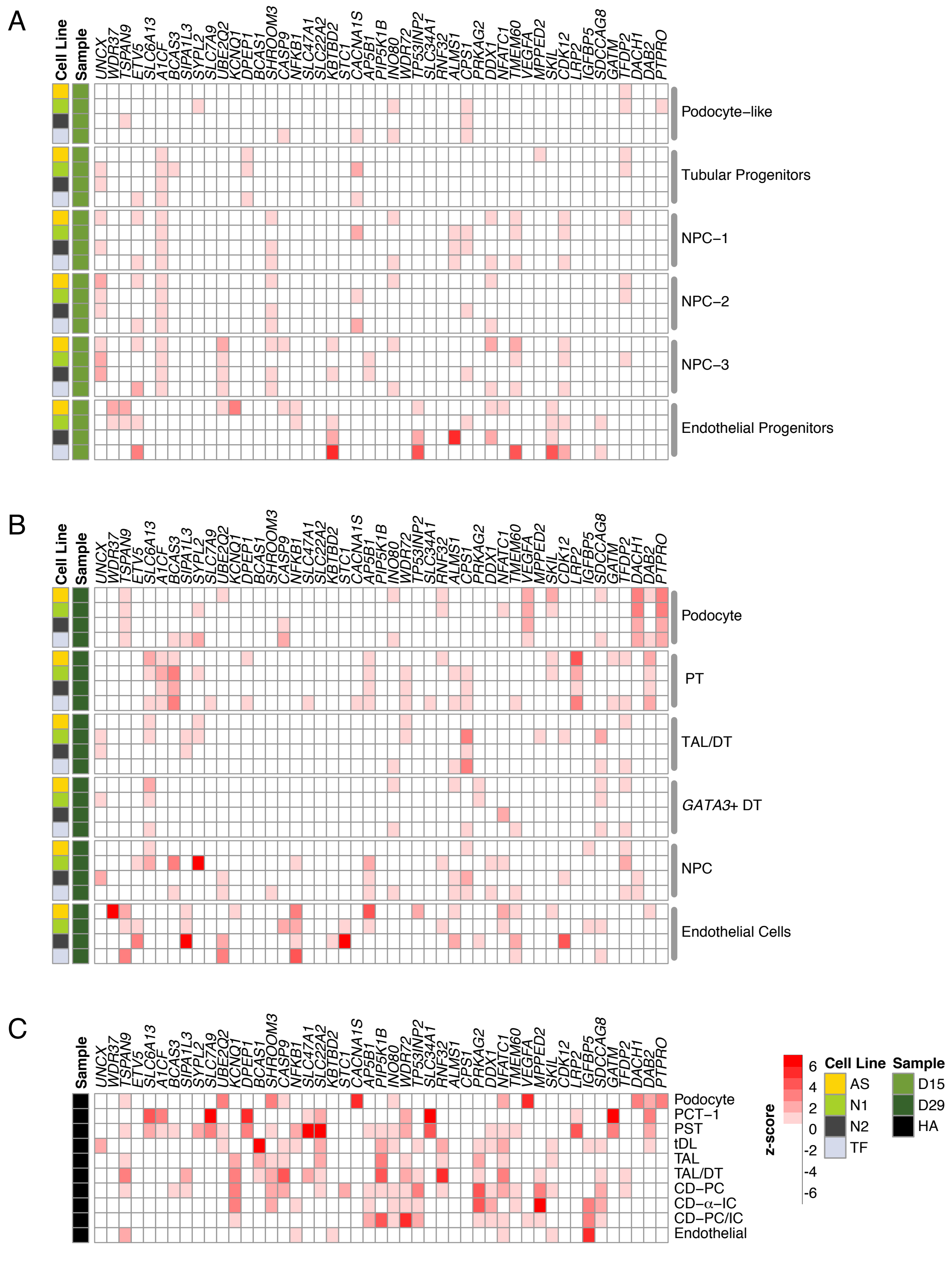
A
B
C
Cell Line
Sample
UNCX
WDR37
TSPAN9
ETV5
SLC6A13
A1CF
BCAS3
SIPA1L3
SYPL2
SLC7A9
UBE2Q2
KCNQ1
DPEP1
BCAS1
SHROOM3
CASP9
NFKB1
SLC47A1
SLC22A2
KBTBD2
STC1
CACNA1S
AP5B1
PIP5K1B
INO80
WDR72
TP53INP2
SLC34A1
RNF32
ALMS1
CPS1
PRKAG2
DDX1
NFATC1
TMEM60
VEGFA
MPPED2
SKIL
CDK12
LRP2
IGFBP5
SDCCAG8
GATM
TFDP2
DACH1
DAB2
PTPRO
Podocyte−like
Tubular Progenitors
NPC−1
NPC−2
NPC−3
Endothelial Progenitors
Podocyte
PT
TAL/DT
GATA3+ DT
NPC
Endothelial Cells
PCT−1
PST
tDL
TAL
CD−PC
CD−α−IC
CD−PC/IC
Endothelial
z-score
6
4
2
0
-2
-4
-6
AS
N1
N2
TF
D15
D29
HA

Supplementary Figure 18. Expression of genes associated with chronic kidney diseases (CKD) in appropriate nephron epithelial cell types suggests utility of kidney organoids for understanding genetic kidney diseases.

Heat map comparison of gene expression in organoids ((A) D15, (B) D29) and (C) human adult of genes associated with chronic kidney disease in kidney epithelial cell clusters across 4 iPSC lines.

Supplementary Figure 19

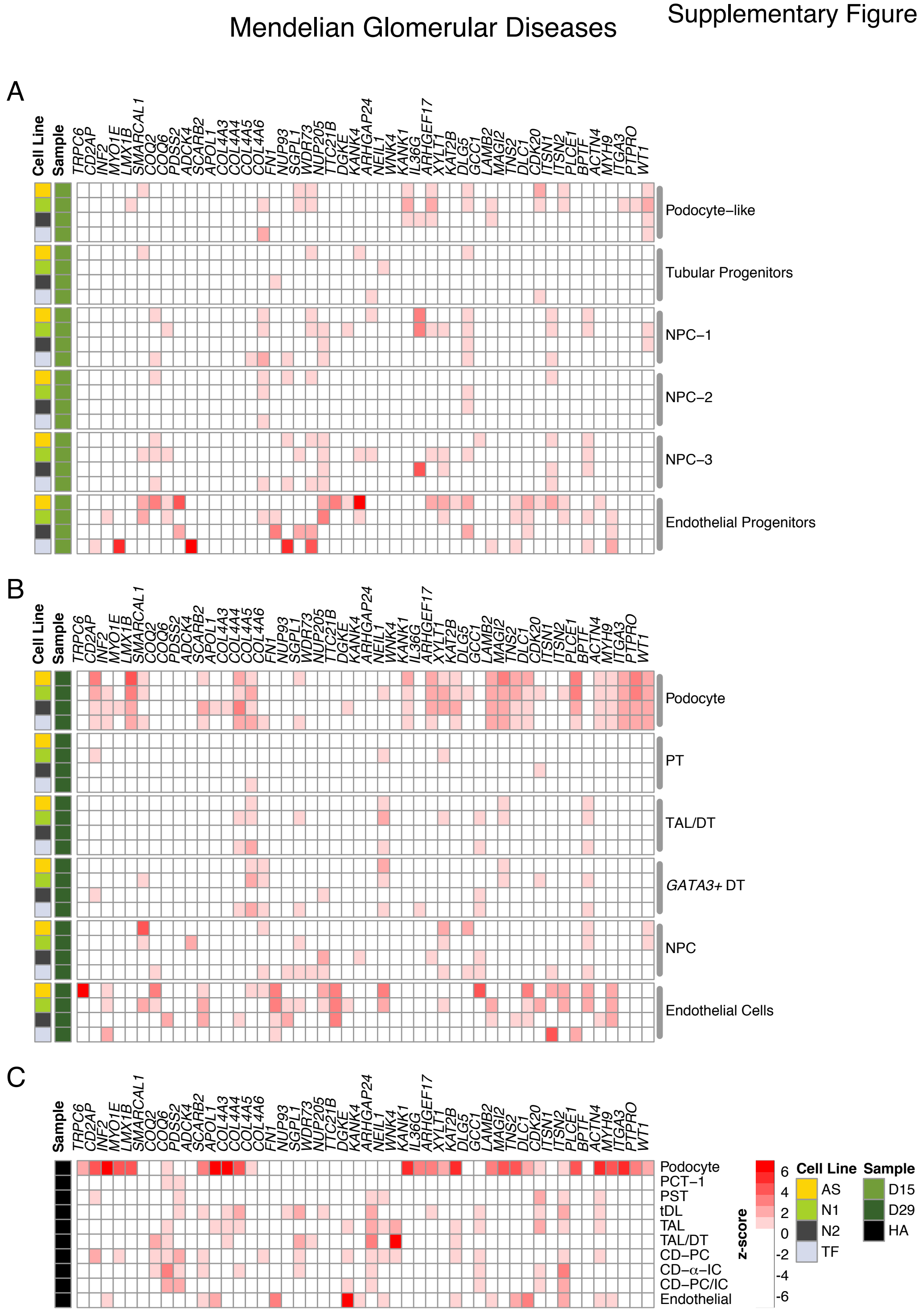

Supplementary Figure 19. Expression of monogenic causes of hereditary glomerular diseases in appropriate nephron epithelial cell types suggests utility of kidney organoids for understanding genetic kidney diseases.
Heat map comparison of gene expression in organoids ((A) D15, (B) D29) and (C) human adult of hereditary glomerular disease-causing genes in kidney epithelial cell clusters across 4 iPSC lines.

Supplementary Figure 20

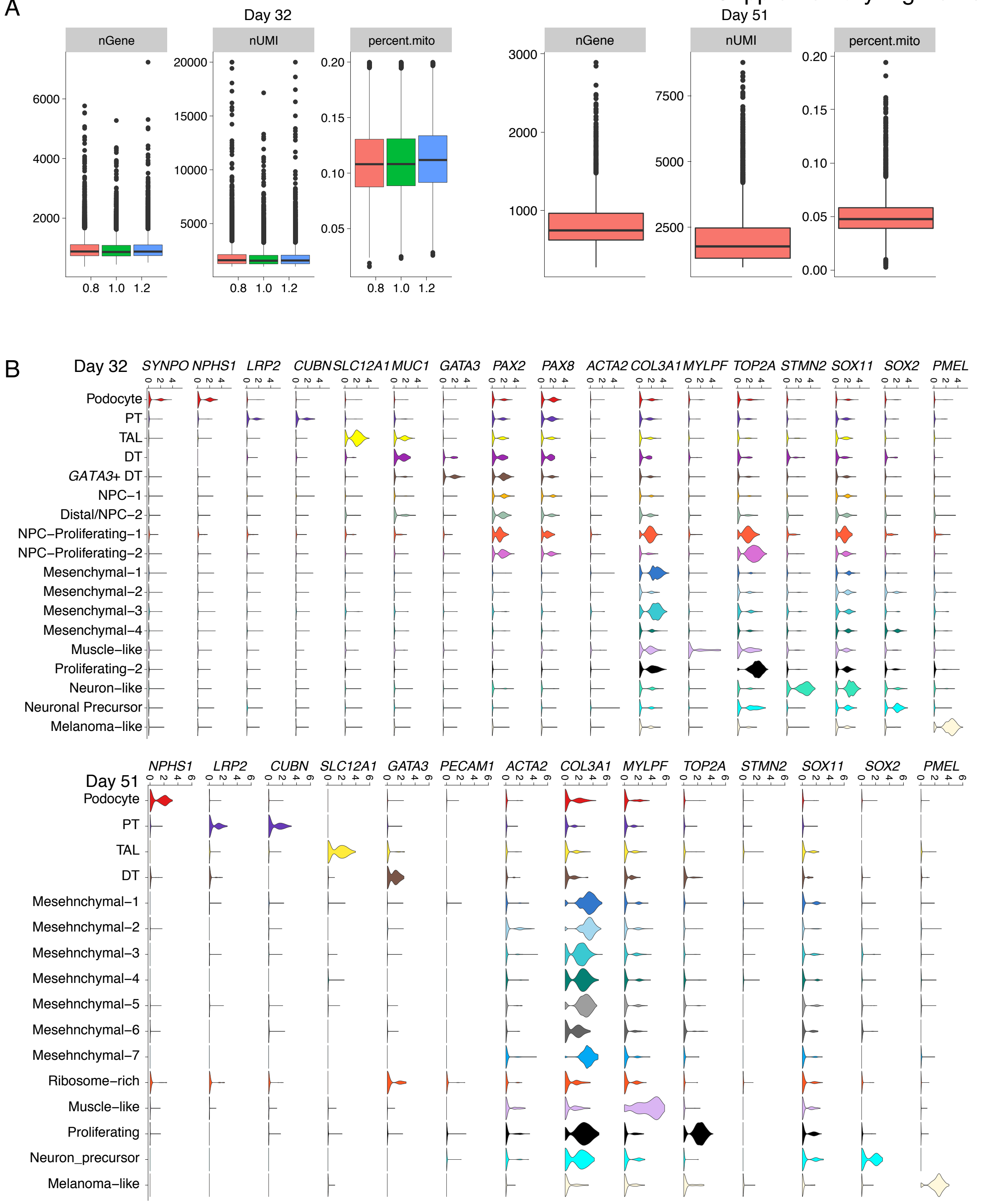

Supplementary Figure 20. Expression of cell type-specific differentially enriched genes in organoids in prolonged culture.

(A) Quality control summaries of D32 and D51 control organoids. The center line of the boxplots indicates the median, and the bottom and top lines of the box indicate the first and third quartiles respectively of the data points. Outliers are indicated as dots beyond the whiskers; whiskers

stretch up to +-1.5*IQR on both sides. (B) Violin plot of single cells from D32 (top row) and D51 (bottom row) control organoids with gene expression of selected genes superimposed.

Supplementary Figure 21

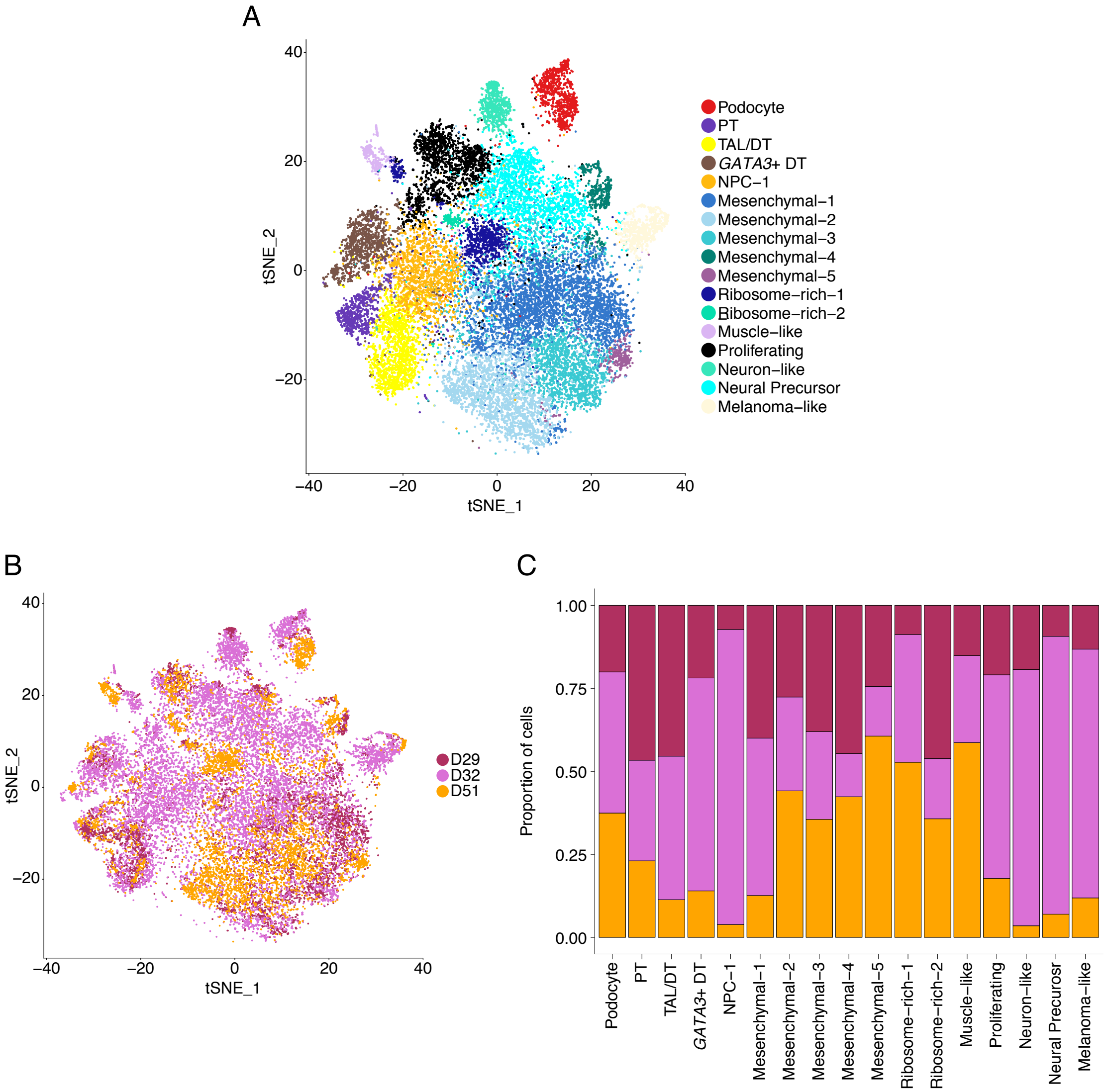

Supplementary Figure 21. Integrated analysis of D29 (ThF), D32, and D51 control kidney organoid data.
(A) t-SNE plot of single cell transcriptomic profiles with cluster color annotations; (B) t-SNE plot of single cell transcriptomic profiles with sample origin color annotations; (C) Proportion plots of sample contribution per cluster.

Supplementary Figure 22

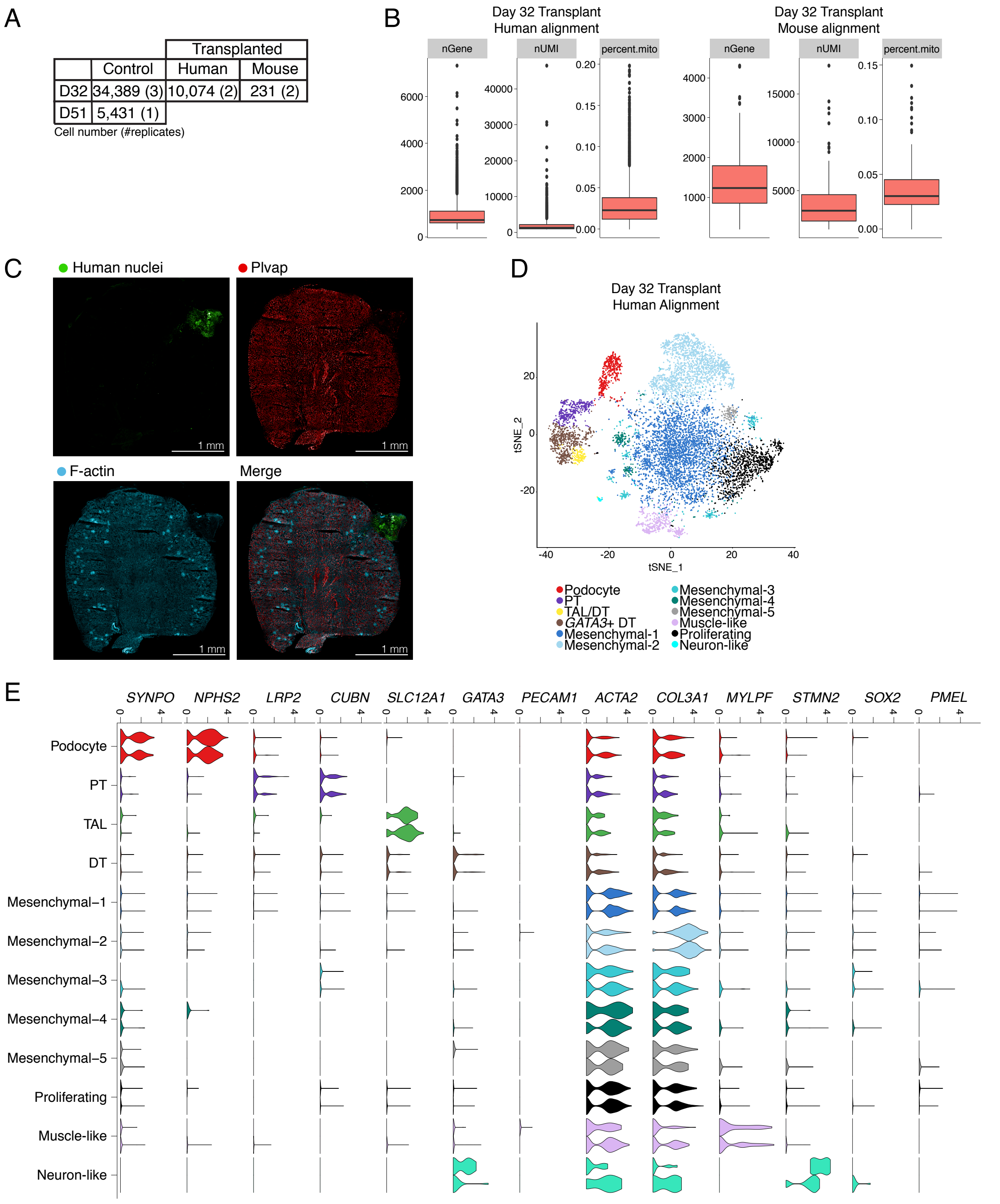

		Transplanted	
	Control	Human	Mouse
D32	34,389 (3)	10,074 (2)	231 (2)
D51	5,431 (1)		

Cell number (#replicates)

Supplementary Figure 22. Single cell analysis of kidney subcapsular transplantation of organoids.

(A) Table of cell numbers from D32 cultured and transplanted organoids. (B) Quality control (QC) summaries of D32 transplanted kidney organoids from alignment to the combined human and mouse transcriptomes for (left) human and (right) mouse cells. The center line of the boxplots indicates the median, and the bottom and top lines of the box indicate the first and third quartiles respectively of the data points. Outliers are indicated as dots beyond the whiskers; whiskers stretch up to +-1.5*IQR on both sides. Data points represent QC summaries as indicated. (C) Immunofluorescence staining of D51 transplanted kidney organoids for human nuclei, mouse endothelial cells (Plvap), and cytoskeleton (F-actin). (D) t-SNE plot of human cells from D32 transplanted kidney organoids. (E) Violin plot of gene expression of canonical cluster markers in single cells from D32 transplanted organoids (human). Y-axis annotations represent clusters. Each violin per cluster represents a transplant replicate.

Supplementary Figure 23

A

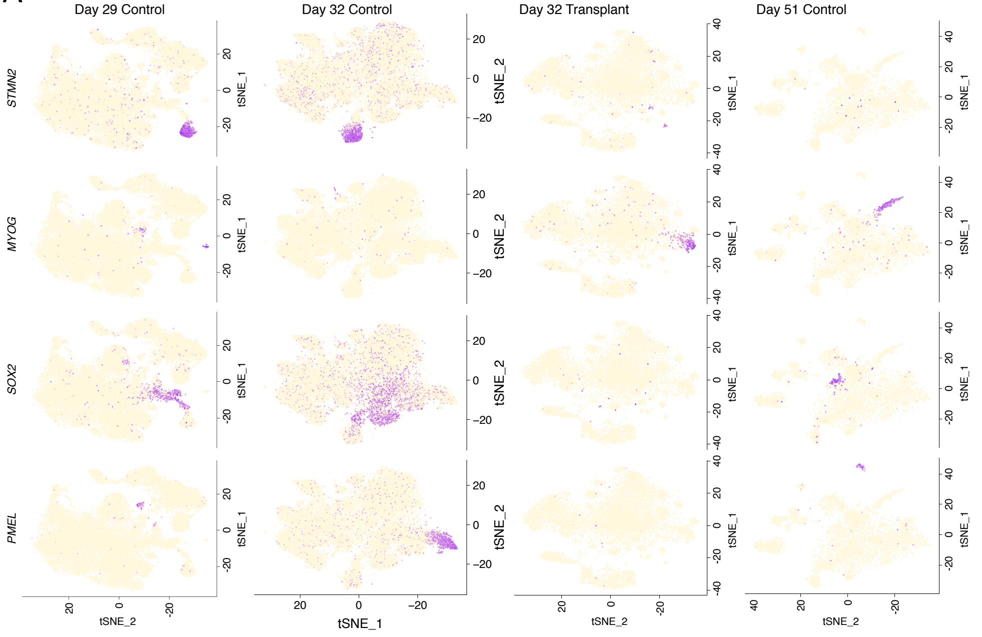

B

	fetal	D29_*SOX2+NTRK2+*	D29_*STMN2+*	D32_*SOX2+NTRK2+*	D32_*STMN2+*	D32_Transplant_*STMN2+*	D51_*SOX2+NTRK2+*
fetal	1	0.33	0.34	0.33	0.41	0.82	0.38
D29_*SOX2+NTRK2+*	0.33	1	0.71	0.94	0.7	0.16	0.67
D29_*STMN2+*	0.34	0.71	1	0.77	0.96	0.26	0.43
D32_*SOX2+NTRK2+*	0.33	0.94	0.77	1	0.79	0.15	0.65
D32_*STMN2+*	0.41	0.7	0.96	0.79	1	0.31	0.48
D32_Transplant_*STMN2+*	0.82	0.16	0.26	0.15	0.31	1	0.15
D51_*SOX2+NTRK2+*	0.38	0.67	0.43	0.65	0.48	0.15	1

Color scale: 1, 0.8, 0.6, 0.4, 0.2, 0, −0.2, −0.4, −0.6, −0.8, −1

Supplementary Figure 23. Off-target *SOX2*+ neuronal- and *PMEL*+ melanoma-like off-target cells reduced in transplanted organoids.
(A) t-SNE plot of single cells from *in vitro* (D29 and D32, and prolonged culture D51) and transplanted organoids with expression of off-target gene markers (*PMEL* [melanoma-like], *SOX2* [neuronal precursor], *MYOG* [muscle-like], *STMN2* [neuronal]). The colors indicate the range of expression from low (off-white) to high (purple). (B) Spearman correlation plot indicates that *STMN2*+ cells in transplanted organoids correlate most (ρ = 0.82) with fetal kidney, in contrast to organoids grown *in vitro*.

Supplementary Figure 24

A

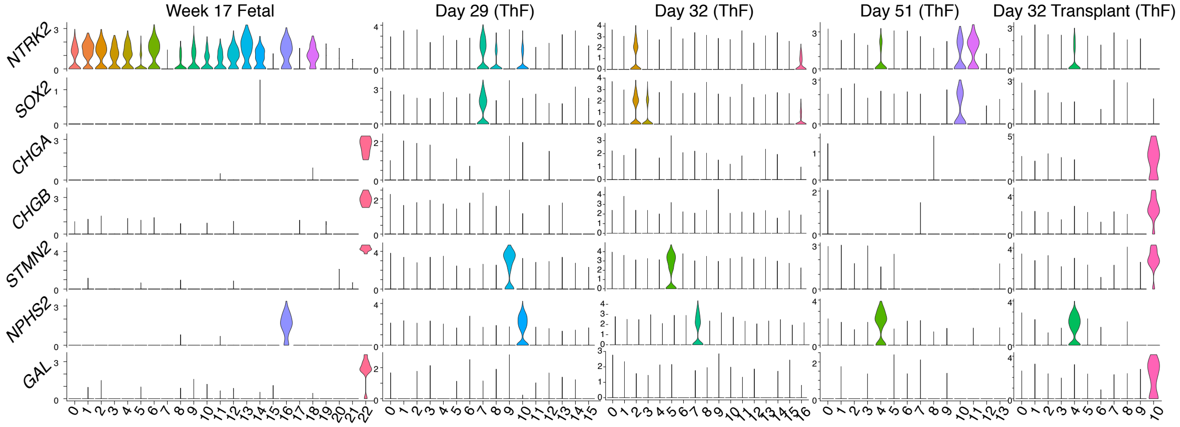

Fetal
0. Mesenchymal-1
1. Mesenchymal-2
2. NPC-prolif
3. Endothelial-precursor
4. Nephron_Progenitor-1
5. Fibroblast-1
6. Nephron_Progenitor-2
7. Endothelial
8. Distal Nephron/UB
9. Fibroblast-2
10. Juxtamedullary
11. Nephron_Progenitor-3
12. Proliferating-1
13. Mesenchymal-3
14. Proliferating-2
15. Immune
16. Podocyte-like
17. Mesenchymal-4
18. Proliferating-3
19. Muscle-like
20. CD-IC
21. Proximal_Nephron
22. Neuron-like

Day 29 (ThF)
0. Mesenchymal_1
1. Neural_Precursor_1
2. Mesenchymal_2
3. TAL_Distal
4. Proliferating
5. PCT
6. *GATA3*+ DT
7. Neural_Precursor_2
8. Mesenchymal_3
9. Neuron_like
10. Podocyte_like
11. Muscle_like
12. Mesenchymal_4
13. Mesenchymal_5
14. Melanoma_like
15. Proliferating-2

Day 32 (ThF)
0. Mesenchymal-1
1. NPC-1
2. Neuronal Precursor
3. Mesenchymal-4
4. *GATA3*+ DT
5. Neuron-like
6. Melanoma-like
7. Podocyte
8. Mesenchymal-2
9. NPC-Proliferating-1
10. TAL
11. Proliferating-2
12. PT/DT
13. Mesenchymal-3
14. Muscle-like
15. Distal/NPC-2
16. NPC-Proliferating-1

Day 51 (ThF)
0. Mesenchymal-1
1. Mesenchymal-2
2. Mesenchymal-3
3. TAL
4. Podocyte
5. Mesenchymal-4
6. Mesenchymal-5
7. Proliferating
8. Muscle-like
9. Mesenchymal-6
10. Neuronal precursor
11. Mesenchymal-7
12. *GATA3*+ DT
13. Melanoma-like

Day 32 Transplant (ThF)
0. Mesenchymal-1
1. Mesenchymal-2
2. Proliferating
3. *GATA3*+ DT
4. Podocyte
5. Muscle-like
6. PT
7. Mesenchymal-3
8. Mesenchymal-4
9. Mesenchymal-5
10. Neuron-like

B

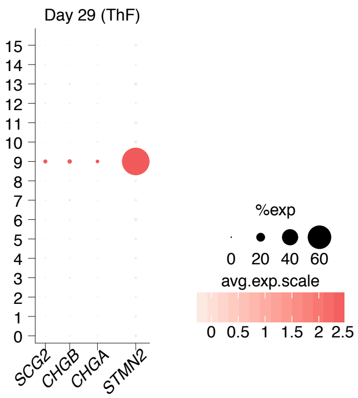

C

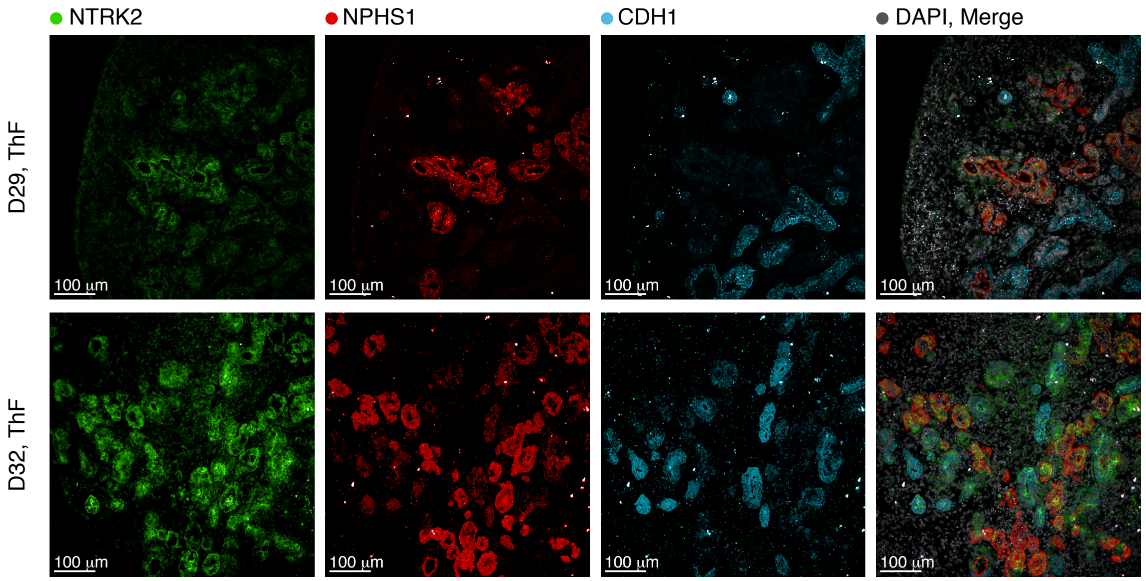

Supplementary Figure 24. Transplanted organoids show similar expression of neuronal genes to second trimester fetal kidneys. (A) Violin plots of clusters from D29 and D32 controls, D51 prolonged culture and D32 transplanted showing expression of specific genes of interest. *NTRK2* is abundantly co-expressed with NPHS2 (podocyte) in fetal kidney and across all organoids. The STMN2+ neuronal cluster in fetal kidney in enriched in CHGA, CHGB and GAL, and this pattern of gene expression is uniquely recapitulated in D32 transplant organoids, but not in organoids grown *in vitro*. (B) Dot plot indicates expression of *CHGA* and *CHGB* was detectable in a small number of cells in D29 *STMN2*+ cells. (C) Immunofluorescence staining of D29 and D32 control kidney organoids for NTRK2, podocytes (NPHS1), and distal tubules (CDH1).

Supplementary Table 1. Table of canonical markers for kidney (organoids and human)

Kidney compartment		Canonical Markers used for identification	
	Organoid	Human	Mouse
Podocyte (1-5)	NPHS2, SYNPO,WT1	NPHS1, NPHS2, SYNPO, WT1, PODXL	
Proximal Tubule (1-4)	CUBN, LRP2,AQP1	CUBN, LRP2,AQP1, SLC7A3, SLC22A7	
Thick Ascending Limb (1-4)	SLC12A1	SLC12A1, UMOD	
Distal Convoluted Tubule (1-4)	-	SLC12A3, MUC1, CDH1	
Distal Nephron (3,4)	CDH1	-	
Ureteric Bud (1,2,7-10)		GATA3, RET, GFRA1, WNT11, ETV4, ETV5	
Collecting Duct – Principal Cells (1-3,6)	-	AQP2, SCNN1B, SCNN1G	
Collecting Duct – Intercalated Cells (1-3,6)		SLC4A9, ATP6V0D2	
Collecting Duct – alpha Intercalated Cells (1-3,6)	-	SLC4A1, KIT	
Collecting Duct – beta Intercalated Cells (1-3,6)	-	SLC26A4, INSRR	
Mesenchymal/vascular smooth muscle	COL3A1, MEIS2, MEIS1 [3,4]	ACTA2 [7]	
Mesenchymal/fibroblast		COL3A1	
Endothelial Cell (1,2,3,5,8,9)	PECAM1	PECAM1, KDR, FLT1, EMCN	Pecam1, Kdr, Flt1, Emcn
Fenestrated Endothelial Cell (10)	-	PECAM1, PLVAP	Pecam1, Plvap
Immune Cells (2)	-	PTPRC	

Other	Organoid
Epithelial (11)	EPCAM
Neuronal (3)	STMN2, SOX11
Neuronal (progenitor) (12,13)	SOX2
Muscle (3)	MYOG, MYLPF
Melanoma-like (3)	PMEL

Other (14-17)	Organoid
Nephron Progenitor Cells (Distal)	POU3F3, PAX2, LHX1, IRX3
Nephron Progenitor Cells (Proximal)	WT1, FOXC2, NOTCH2

Supplementary Table 2. Table of data-driven markers from D29 and D15

Organoid Stage	Kidney Cell	Data-driven Marker
D15	Podocyte	CLDN5
		SOST
		SPARC
		BST2
D29	Proximal Tubule (Proximal-distal gradient)	APOE
	Distal Nephron	WFDC2
		MAL
		DEFB1

Supplementary Table 3. Table of cell types and compartments

Putative_Cell_Types	Compartment
Endothelial Cells	Endothelial
Distal Nephron	Nephron
Nephron Progenitor Cells	Nephron
Podocyte	Nephron
Proximal Convoluted Tubule	Nephron
Thick Ascending Limb	Nephron
Mesenchymal Cells-1	Mesenchymal
Mesenchymal Cells-2	Mesenchymal
Mesenchymal Cells-3	Mesenchymal
Mesenchymal Cells-4	Mesenchymal
Mesenchymal Cells-5	Mesenchymal
Mesenchymal Cells-6	Mesenchymal
Mesenchymal Cells-7	Mesenchymal
Mesenchymal Cells-8	Mesenchymal
Proliferating	Mesenchymal
Muscle-like-1	Offtarget
Muscle-like-2	Offtarget
Neuron-like	Offtarget
Neuronal precursor	Offtarget
Melanoma	Offtarget

Supplementary Table 4. Table of developmental programs (14-17).

**Ureteric Bud**	RET, WNT9B, LHX1, ETV4, ETV5, WNT11, SPRY1, FGF9, GATA3, GFR1A
**Metanephric Mesenchyme**	OSR1, SALL1, WT1, EYA1, HOXD11, CITED1, PAX2, SIX1, SIX2, GDNF
**Cap Mesenchyme**	GDNF, PAX2, WT1, SIX2, CITED1, EYA1, WNT4
**Pretubular aggregate**	WNT4, LHX1
**Renal Vesicle – distal**	PAX2, LHX1, IRX3 (BRN1), LHX1
**Renal Vesicle – Proximal**	NOTCH2
**Renal Vesicle – Glomerulus**	FOXC2, WT1, VEGF

Supplemental References

1. Park, J. *et al.* Single-cell transcriptomics of the mouse kidney reveals potential cellular targets of kidney disease. *Science (80-.).* **360**, 758–763 (2018).
2. Wu, H., Kirita, Y., Donnelly, E. L. & Humphreys, B. D. Advantages of Single-Nucleus over Single-Cell RNA Sequencing of Adult Kidney: Rare Cell Types and Novel Cell States Revealed in Fibrosis. *J. Am. Soc. Nephrol.* **30**, 23–32 (2019).
3. Wu, H. *et al.* Comparative Analysis and Refinement of Human PSC-Derived Kidney Organoid Differentiation with Single-Cell Transcriptomics. *Cell Stem Cell* **23**, 869-881.e8 (2018).
4. Takasato, M. *et al.* Kidney organoids from human iPS cells contain multiple lineages and model human nephrogenesis. *Nature* **526**, 564–568 (2015).
5. Karaiskos, N. *et al.* A Single-Cell Transcriptome Atlas of the Mouse Glomerulus. *J. Am. Soc. Nephrol.* **29**, 2060–2068 (2018).
6. Chen, L. *et al.* Transcriptomes of major renal collecting duct cell types in mouse identified by single-cell RNA-seq. *Proc. Natl. Acad. Sci.* **114**, E9989–E9998 (2017).
7. Xie, T. *et al.* Single-Cell Deconvolution of Fibroblast Heterogeneity in Mouse Pulmonary Fibrosis. *Cell Rep.* **22**, 3625–3640 (2018).
8. Lukowski, S. W. *et al.* Single-Cell Transcriptional Profiling of Aortic Endothelium Identifies a Hierarchy from Endovascular Progenitors to Differentiated Cells. *Cell Rep.* **27**, 2748-2758.e3 (2019).
9. Kalluri, A. S. *et al.* Single Cell Analysis of the Normal Mouse Aorta Reveals Functionally Distinct Endothelial Cell Populations. *Circulation* CIRCULATIONAHA.118.038362 (2019). doi:10.1161/CIRCULATIONAHA.118.038362
10. Stan, R. V. *et al.* The Diaphragms of Fenestrated Endothelia: Gatekeepers of Vascular Permeability and Blood Composition. *Dev. Cell* **23**, 1203–1218 (2012).
11. Phipson, B. *et al.* Evaluation of variability in human kidney organoids. *Nat. Methods* **16**, 79–87 (2019).
12. Graham, V., Khudyakov, J., Ellis, P. & Pevny, L. SOX2 functions to maintain neural progenitor identity. *Neuron* **39**, 749–765 (2003).
13. Ellis, P. *et al.* SOX2, a persistent marker for multipotential neural stem cells derived from embryonic stem cells, the embryo or the adult. *Dev. Neurosci.* **26**, 148–65
14. Little, M. H. & McMahon, A. P. Mammalian kidney development: Principles, progress, and projections. *Cold Spring Harb. Perspect. Biol.* **4**, 3 (2012).
15. Sharma, R., Sanchez-Ferras, O. & Bouchard, M. Pax genes in renal development, disease and regeneration. *Semin. Cell Dev. Biol.* **44**, 97–106 (2015).
16. Sirin, Y. & Susztak, K. Notch in the kidney: development and disease. *J. Pathol.* **226**, 394–403 (2012).
17. Krause, M., Rak-Raszewska, A., Pietilä, I., Quaggin, S. & Vainio, S. Signaling during Kidney Development. *Cells* **4**, 112–132 (2015).
18. Akchurin, O. & Reidy, K. J. Genetic causes of proteinuria and nephrotic syndrome: Impact on podocyte pathobiology. *Pediatr. Nephrol.* **30**, 221–233 (2015).
19. Wenderfer, S. E. & Gaut, J. P. Glomerular Diseases in Children. *Adv. Chronic Kidney Dis.* **24**, 364–371 (2017).
20. Dillman, J. R., Trout, A. T., Smith, E. A. & Towbin, A. J. Hereditary Renal Cystic Disorders: Imaging of the Kidneys and Beyond. *Radiographics* **37**, 924–946 (2017).
21. Capone, V. P., Morello, W., Taroni, F. & Montini, G. Genetics of Congenital Anomalies of the Kidney and Urinary Tract: The Current State of Play. *Int. J. Mol. Sci.* **18**, (2017).
22. Vivante, A. & Hildebrandt, F. Exploring the genetic basis of early-onset chronic kidney disease. *Nat. Rev. Nephrol.* **12**, 133–146 (2016).
23. Ware, S. M., Aygun, M. G.- & Hildebrandt, F. Spectrum of Clinical Diseases Caused By Disorders of Primary Cilia. *Proc. Am. Thorac. Soc.* **8**, 444–450 (2011).

24. Hildebrandt, F. Genetic kidney diseases. *Lancet (London, England)* **375**, 1287–95 (2010).
25. Warejko, J. K. *et al.* Whole exome sequencing of patients with steroid-resistant nephrotic syndrome. *Clin. J. Am. Soc. Nephrol.* **13**, 53–62 (2018).
26. Brown, E. J., Pollak, M. R. & Barua, M. Genetic testing for nephrotic syndrome and FSGS in the era of next-generation sequencing. *Kidney Int.* **85**, 1030–1038 (2014).
27. Yu, H. *et al.* A role for genetic susceptibility in sporadic focal segmental glomerulosclerosis. *J. Clin. Invest.* **126**, 1067–1078 (2016).
28. Ashraf, S. *et al.* Mutations in six nephrosis genes delineate a pathogenic pathway amenable to treatment. *Nat. Commun.* **9**, 1960 (2018).
