# Supplementary material for: Single cell census of human kidney organoids shows reproducibility and diminished off-target cells after transplantation
Source: Nat Commun. 2019 Nov 29;10:5462. doi: 10.1038/s41467-019-13382-0 (PMC6884507; doi:10.1038/s41467-019-13382-0)
Supplement: Supplementary file 3 — Description of Additional Supplementary Files [file 41467_2019_13382_MOESM3_ESM.pdf]

Description of Additional Supplementary Files

Supplementary Data 1. Cluster gene lists for D29 after batch-correction (representative line: ThF)
Supplementary Data 2. Table of cell-cycle genes
Supplementary Data 3. Random forest gene lists
Supplementary Data 4: Cluster gene lists for iPSC after batch-correction (representative line: AS)
Supplementary Data 5. Table of germ layer signatures
Supplementary Data 6. Cluster gene lists for D7 after batch-correction (representative line: AS)
Supplementary Data 7. Cluster gene lists for D15 after batch-correction (representative line: ThF)
Supplementary Data 8. Cluster gene lists for joint D0,D7, D15 after batch-correction (representative line: ThF)
Supplementary Data 9. Table of kidney disease genes with references
Supplementary Data 10. Cluster gene lists for joint D29, D32, D51 after batch-correction (line: ThF)
Supplementary Data 11. Cluster gene lists for D32 transplanted organoid (line: ThF)
