# Supplementary material for: Single cell census of human kidney organoids shows reproducibility and diminished off-target cells after transplantation
Source: Nat Commun. 2019 Nov 29;10:5462. doi: 10.1038/s41467-019-13382-0 (PMC6884507; doi:10.1038/s41467-019-13382-0)
Supplement: Supplementary file 15 — Reporting Summary [file 41467_2019_13382_MOESM15_ESM.pdf]

nature research

Corresponding author(s): Anna Greka

Last updated by author(s): Oct 28, 2019

Reporting Summary

Nature Research wishes to improve the reproducibility of the work that we publish. This form provides structure for consistency and transparency in reporting. For further information on Nature Research policies, see Authors & Referees and the Editorial Policy Checklist.

Statistics

For all statistical analyses, confirm that the following items are present in the figure legend, table legend, main text, or Methods section.

n/a	Confirmed	
☐	☒	The exact sample size (*n*) for each experimental group/condition, given as a discrete number and unit of measurement
☐	☒	A statement on whether measurements were taken from distinct samples or whether the same sample was measured repeatedly
☐	☒	The statistical test(s) used AND whether they are one- or two-sided<br>*Only common tests should be described solely by name; describe more complex techniques in the Methods section.*
☐	☒	A description of all covariates tested
☐	☒	A description of any assumptions or corrections, such as tests of normality and adjustment for multiple comparisons
☐	☒	A full description of the statistical parameters including central tendency (e.g. means) or other basic estimates (e.g. regression coefficient) AND variation (e.g. standard deviation) or associated estimates of uncertainty (e.g. confidence intervals)
☐	☒	For null hypothesis testing, the test statistic (e.g. *F*, *t*, *r*) with confidence intervals, effect sizes, degrees of freedom and *P* value noted<br>*Give P values as exact values whenever suitable.*
☒	☐	For Bayesian analysis, information on the choice of priors and Markov chain Monte Carlo settings
☒	☐	For hierarchical and complex designs, identification of the appropriate level for tests and full reporting of outcomes
☐	☒	Estimates of effect sizes (e.g. Cohen's *d*, Pearson's *r*), indicating how they were calculated

Our web collection on statistics for biologists contains articles on many of the points above.

Software and code

Policy information about availability of computer code

Data collection	Public dataset (GSE112570) was downloaded from GEO using the "wget" command. The supplementary tables from PMID: 26501952 (nbt.3387-S5.xlsx) and PMID:30093597 (Supplementary Data Table) were downloaded for germ layer signatures and trimester 1 fetal kidney data respectively.
Data analysis	Demultiplexing and quantification of sequencing files was performed using 10X's CellRanger pipeline (v2.1.1) . All analysis was performed using the R statistical computing language (v3.4.1). We used the default settings in the Seurat R package (v2.3) for single-cell data analysis. The R package ggplot was used for visualization of tSNEs, cell-type proportions and dotplots. The R package Randomforest was used for classification-based analysis. The philentropy package was used for testing compositional differences.

For manuscripts utilizing custom algorithms or software that are central to the research but not yet described in published literature, software must be made available to editors/reviewers. We strongly encourage code deposition in a community repository (e.g. GitHub). See the Nature Research guidelines for submitting code & software for further information.

Data

Policy information about availability of data

All manuscripts must include a data availability statement. This statement should provide the following information, where applicable:

- Accession codes, unique identifiers, or web links for publicly available datasets
- A list of figures that have associated raw data
- A description of any restrictions on data availability

The germ-layer signatures were obtained from Tsankov et al. 2015 (PMID: 26501952, nbt.3387-S5.xlsx). Public Dataset (NCBI GEO GSE112570) used for human fetal data (Trimester 2) analysis. Trimester 1 human fetal kidney single-cell transcriptomes were downloaded from the Data Supplement in Young et al., 2018(PMID:30093597). In both cases, the data were available in the format of gene expression count matrices.

The sequencing data that support the findings of this study have been deposited at GEO (Accession number GSE136314) and will also be available from the Human Cell Atlas portal (https://preview.data.humancellatlas.org/).

Field-specific reporting

Please select the one below that is the best fit for your research. If you are not sure, read the appropriate sections before making your selection.

☒ Life sciences ☐ Behavioural & social sciences ☐ Ecological, evolutionary & environmental sciences

For a reference copy of the document with all sections, see nature.com/documents/nr-reporting-summary-flat.pdf

Life sciences study design

All studies must disclose on these points even when the disclosure is negative.

Sample size	Sample-size calculation was not explicitly performed, and sample sizes were chosen to allow replicates and reproducibility of results. In line with Supp Fig 1A, we had 49 kidney organoid states in total. The organoids were derived from 4 donor lines to account for donor-variability, and from 3 different stem cell facilities. 2 of the donors were male and 2 female to account for any sexual dimorphism. The iPSCs were reprogrammed using different protocols (Sendai virus or Episomal) to account for any differences arising from reprogramming protocols. For each of the iPSC-derived organoids, 3 organoid clones were sequenced at the mature and Day 15 stage. For one iPSC (ThF), we included two differentiation experiments to allow for experiment-experiment variability.
Data exclusions	Quality control was performed on the single-cell data count matrix as described in the Methods section to exclude low-quality cells from downstream analysis.
Replication	3 organoid clones for each line at time-points 15 and 29. Replicates from both sexes were incorporated wherever possible. A subset of discovered markers were validated using immunofluorescence. All representative images reflect a minimum of three biological replicates.
Randomization	Organoids were pooled on lanes using a randomized design to ensure that organoids replicates from an individual batch (donor, replicate, condition) were distributed across lanes.
Blinding	Blinding was not possible and not relevant to the study as the goal was to examine reproducibility among cell-lines from different iPSCs, a piece of information that we had to explicitly account for.

Reporting for specific materials, systems and methods

We require information from authors about some types of materials, experimental systems and methods used in many studies. Here, indicate whether each material, system or method listed is relevant to your study. If you are not sure if a list item applies to your research, read the appropriate section before selecting a response.

Materials & experimental systems

n/a	Involved in the study	
☐	☒	Antibodies
☐	☒	Eukaryotic cell lines
☒	☐	Palaeontology
☐	☒	Animals and other organisms
☐	☒	Human research participants
☒	☐	Clinical data

Methods

n/a	Involved in the study	
☒	☐	ChIP-seq
☒	☐	Flow cytometry
☒	☐	MRI-based neuroimaging

Antibodies

Antibodies used

WT1 (ThermoFisher, no. PA5-16879, 1:100), RRID:AB_10979199
ECAD, ECAD (Abcam, no. ab11512, 1:500 RRID:AB_298118
SYNPO, (Abcam, no. ab117702, 1:500) RRID:AB_10899584
MUC1, (Abcam, no. ab80952, 1:500) RRID:AB_1640314
Fluorescein labeled LTL (Vector Laboratories, no. FL-1321, 1:300), RRID:AB_2336559
GATA3 (Cell Signaling Technology, no. 5852, 1:300) RRID:AB_10835690
SOX2 (Cell Signaling Technology, no. 3579, 1:300) RRID:AB_2195767
Laminin (Sigma-Aldrich, no. L9393, 1:500), RRID:AB_477163
MEIS1 (Activemotif, no. ATM39795, 1:300) RRID:AB_2750570
CD31 (BD Pharmingen,no. 555444, 1:300), RRID:AB_395837
SOX17 (R&D Systems, no. AF1924, 1:300), RRID:AB_355060
LRP2 (Santa Cruz Biotechnology, no. 515772, 1:100), RRID:AB_2783023
PAX2 (Zymed laboratories, no. 71-6000, 1:300). RRID:AB_2533990
Human nuclei (Antibodies online, no. ABIN361360, 1:300), RRID:AB_10774181
MECA-32 (BD Biosciences, no. 553849, 1:300), RRID:AB_395086

	NPHS1 (R&D systems, no. AF4269, 1:300) RRID:AB_2154851<br>Claudin-5 Antibody (Novus Biologicals, no.NBP2-66783, 1:300). RRID:AB_2783024<br>NTRK2 Antibody (abclonal A2099, 1:300) RRID:AB_2764118<br>All Alexa Fluor secondary antibodies were purchased from Thermo Fisher Scientific (1:1000).<br>Donkey anti-Rat IgG (H+L) Highly Cross-Adsorbed Secondary Antibody, Alexa Fluor 488 (Thermo Fisher Scientific Cat# A-21208, 1:1000) RRID:AB_2535794<br>Donkey anti-Sheep IgG (H+L) Cross-Adsorbed Secondary Antibody, Alexa Fluor 568. (Thermo Fisher Scientific Cat# A-21099, 1:1000) RRID:AB_2535753<br>Donkey anti-Rabbit IgG (H+L) Highly Cross-Adsorbed Secondary Antibody, Alexa Fluor 568 (Thermo Fisher Scientific Cat# A10042, 1:1000) RRID:AB_2534017<br>Donkey anti-Mouse IgG (H+L) Highly Cross-Adsorbed Secondary Antibody, Alexa Fluor 568 (Thermo Fisher Scientific Cat# A10037, 1:1000) RRID:AB_2534013<br>Goat anti-Mouse IgM (Heavy chain) Cross-Adsorbed Secondary Antibody, Alexa Fluor 488 (Thermo Fisher Scientific Cat# A-21042, 1:1000) RRID:AB_2535711<br>Goat anti-Rabbit IgG (H+L) Cross-Adsorbed Secondary Antibody, Alexa Fluor 405 (Thermo Fisher Scientific Cat# A-31556, 1:1000) RRID:AB_221605<br>Donkey anti-Rat IgG (H+L) Highly Cross-Adsorbed Secondary Antibody, Alexa Fluor 488 (Thermo Fisher Scientific Cat# A-21208, 1:1000) RRID:AB_2535794<br>Goat anti-Rat IgG (H+L) Cross-Adsorbed Secondary Antibody, Alexa Fluor 647 (Thermo Fisher Scientific Cat# A-21247, 1:1000) RRID:AB_141778
Validation	All the antibodies are validated for Immunofluorescence (IF) in human kidney organoids

Eukaryotic cell lines

Policy information about cell lines

Cell line source(s)	Human Episomal iPSC Line (ThermoFisher, no. A18945, ALSTEM, no. iPS16). N1 line (S1930 CB A) N2 line (S1973 WR I) were derived from erythroblasts using CTS™ CytoTune™-iPS 2.1 Sendai Reprogramming Kit (ThermoFisher, no. A34546) at Harvard Stem Cell Institute (HSCI) iPS Core Facility
Authentication	The N1 and N2 cell lines were characterized for pluripotency and spontaneous differentiation to the three germ layers using qPCR based on standard protocols at the HSCI Core Facility.
Mycoplasma contamination	All lines were confirmed to be karyotype normal and maintained below passage 15 and all the cell lines were routinely checked and were negative for mycoplasma
Commonly misidentified lines (See ICLAC register)	No misidentified cell lines were used

Animals and other organisms

Policy information about studies involving animals; ARRIVE guidelines recommended for reporting animal research

Laboratory animals	Female mice, NOD scid gamma (NSG) 8-weekold, The Jackson Laboratory, no. Jax # 005557).
Wild animals	No wild animals were involved in the study
Field-collected samples	No field collected samples were used in the study.
Ethics oversight	Animal experiments were done at Custom contract research company Biomere (Biomedical Research Model company (https://biomere.com). Biomere has all the IACUC approval for animal experiments.

Note that full information on the approval of the study protocol must also be provided in the manuscript.

Human research participants

Policy information about studies involving human research participants

Population characteristics	Human tissue was obtained from nephrectomies from patients with normal kidney function. The tissue was then immediately stored on ice (in RPMI medium supplemented with 2% heat inactivated fetal bovine serum and penicillin/streptomycin) until tissue processing commenced in our laboratory (within ~45 minutes).
Recruitment	3 Samples were collected on this radical nephrectomy for tumor resection on a random adult patients without history of medical renal disease at Brigham and Women's Hospital, provided that enough grossly unremarkable renal parenchyma could be identified post resection, which could be allocated without compromising diagnosis or specimen integrity. No specific patient recruitment was performed. No specific patient consent was necessary per IRB protocol 2011P002692. A section of uninvolved renal parenchyma adjacent to the allocation site was routinely processed for histologic analysis by a renal pathologist.

Ethics oversight | BWH Institutional Review Board, approved Protocol Number 2011P002692

Note that full information on the approval of the study protocol must also be provided in the manuscript.
