# Supplementary material for: Single cell census of human kidney organoids shows reproducibility and diminished off-target cells after transplantation
Source: Nat Commun. 2019 Nov 29;10:5462. doi: 10.1038/s41467-019-13382-0 (PMC6884507; doi:10.1038/s41467-019-13382-0)
Supplement: Supplementary file 2 — Peer Review File [file 41467_2019_13382_MOESM2_ESM.pdf]

Peer Review File

Editorial Note: This manuscript has been previously reviewed at another journal that is not operating a transparent peer review scheme. This document only contains reviewer comments and rebuttal letters for versions considered at *Nature Communications.*
